# “DNA Methylation signatures in panic disorder”

**DOI:** 10.1038/s41398-017-0026-1

**Published:** 2017-12-18

**Authors:** Stella Iurato, Tania Carrillo-Roa, Janine Arloth, Darina Czamara, Laura Diener-Hölzl, Jennifer Lange, Bertram Müller-Myhsok, Elisabeth B. Binder, Angelika Erhardt

**Affiliations:** 1 Department of Translational Research in Psychiatry, Max Planck Institute of Psychiatry, Munich, Germany; 2 Department of Psychiatry and Behavioral Sciences, Emory University, Atlanta, GA USA

## Abstract

Panic disorder (PD) affects about four million Europeans, with women affected twice as likely as men, causing substantial suffering and high economic costs. The etiopathogenesis of PD remains largely unknown, but both genetic and environmental factors contribute to risk. An epigenome-wide association study (EWAS) was conducted to compare medication-free PD patients (*n* = 89) with healthy controls (*n* = 76) stratified by gender. Replication was sought in an independent sample (131 cases, 169 controls) and functional analyses were conducted in a third sample (*N* = 71). DNA methylation was assessed in whole blood using the Infinium HumanMethylation450 BeadChip. One genome-wide association surviving FDR of 5% (cg07308824, *P* = 1.094 × 10-7, P-adj = 0.046) was identified in female PD patients (*N* = 49) compared to controls (*N* = 48). The same locus, located in an enhancer region of the *HECA* gene, was also hypermethylated in female PD patients in the replication sample (*P* = 0.035) and the significance of the association improved in the meta-analysis (P-adj = 0.004). Methylation at this CpG site was associated with *HECA* mRNA expression in another independent female sample (*N* = 71) both at baseline (*P* = 0.046) and after induction by dexamethasone (*P* = 0.029). Of 15 candidates, 5 previously reported as associated with PD or anxiety traits also showed differences in DNA methylation after gene-wise correction and included *SGK1*, *FHIT*, *ADCYAP1*, *HTR1A*, *HTR2A*. Our study examines epigenome-wide differences in peripheral blood for PD patients. Our results point to possible sex-specific methylation changes in the *HECA* gene for PD but overall highlight that this disorder is not associated with extensive changes in DNA methylation in peripheral blood.

## Introduction

Panic disorder (PD) is the most disabling anxiety disorder, causing substantial suffering, and high economic and social costs. It affects about four million Europeans (12-month prevalence estimate is about 2%) with women being twice as likely to be affected as men^1^. PD is characterized by sudden episodes of acute anxiety (panic attacks) occurring without any apparent reason. It can be accompanied by a persistent concern of having additional attacks or worry about the possible consequences of the attacks (e.g., suffering of a heart attack, dying, losing control) and significant behavioral changes to avoid future panic attacks^2^. First onset for PD is in adolescence and early adulthood^2^ and it is highly co-morbid with other mental disorders, especially agoraphobia^3^.

Despite the substantial long-term disability, PD appears to be underdiagnosed and undertreated in mental health settings. The overall heritability of PD is substantial with heritability estimates up to 48%^4^. Genetic studies (genome-wide as well as candidate gene approaches) have identified some loci possibly contributing to disease risk such as variants in the *TMEM132D* locus^5, 6^, *COMT* ^7^, *CRHR1* ^8^, *SLC6A4*, *MAOA*, *HTR1A* ^9^, but overall genetic linkage or association signals are usually not consistently replicated.

Exposure to negative life events are the complementing risk factors to genetics so that both genetic and environmental components are likely important mechanisms influencing disease risk^10^. The differential contribution of environmental and genetic factors in risk for anxiety disorders, personality disorders, alcohol use disorders has been previously supported by epidemiologic and twin studies^11–13^. Adverse life events have been shown to associate with specific epigenetic modifications, such as DNA methylation, which may mediate the lasting cellular consequences of these exposures in psychiatric disorders^14^, also in the context of gene–environment interactions^15^. Exploring DNA methylation may thus be able to give an integrated view of both environmental and genetic risk factors. While epigenetic changes including DNA methylation are mainly tissue specific, some sites show cross tissue relevance^16, 17^ and furthermore changes in peripheral tissues such as blood could serve as potential biomarker for disease risk.

To date, a few studies have investigated differences in DNA methylation in candidate genes in PD^5, 18–21^. Recently, one epigenome-wide study of DNA methylation in a relatively small Japanese PD case-control sample (48 patients vs. 48 controls) detected significant associations at 40 sites with overall small methylation differences^22^. So far, studies in the European population are lacking. To perform such a study in two independent samples of patients with PD vs. control was the aim of our study. To reduce confounding due to effects of drug treatment, both patients and controls were free of psychotropic medication. Given that both the prevalence of PD as well as DNA methylation pattern show large gender differences^23^, a gender-stratified analysis was undertaken and complemented by a meta-analysis.

Previous studies report that hits identified in genome-wide association studies (GWAS) show changes in DNA methylation in peripheral blood, e.g., in schizophrenia^24^ or bipolar disorder^25^. For this reason, in addition to an unbiased approach, we also investigated DNA methylation changes in candidate genes that have emerged from genome-wide or candidate gene studies for PD, anxiety disorders or anxiety related phenotypes either in humans or animals^5,26, 27^.

PD has a high comorbidity rate not only with other psychiatric disorders like agoraphobia and depression, but also with other medical conditions, e.g., cardiovascular disorders, asthma and epilepsy^1^. DNA methylation age has been previously correlated with morbidity and mortality^28–31^. Therefore, we also investigated whether age acceleration was occurring in PD patients compared to controls.

## Materials and Methods

### Max Planck Institute of Psychiatry PD cohort

PD patients included in the discovery and replication sample were recruited in the anxiety disorders outpatient unit at the MPIP in Munich^5^. PD was the primary diagnosis; mild secondary depression was allowed (Table 1). The diagnosis was ascertained by trained psychiatrists according to the Diagnostic and Statistical Manual of Mental Disorders (DSM)-IV criteria. All patients underwent the Structured Clinical Interviews for DSM-IV (SCID I and II). PD due to a medical or neurological condition or the presence of a comorbid Axis II disorder was an exclusion criterion. All patients underwent a thorough medical examination including EEG, ECG and detailed hormone laboratory assessment.

**Table 1:** Characteristics of the participants included in the study

Variable	Controls	Cases	Total
*Discovery*			
Participants, *N* (%)	76 (46%)	89 (54%)	165
Male, *N* (%)	28	40	68 (41%)
Female, *N* (%)	48	49	97 (59%)
Age, years (SD)	37 (7.5)	36 (10.4)	
Diagnosis	None	PDA 72%	
		PD 28%	
		Comorbidity:MDD 13.5%	
*Replication*			
Participants, *N* (%)	169 (56%)	131 (44%)	300
Male, *N* (%)	48	48	96 (32%)
Female, *N* (%)	121	83	204 (68%)
Age, years (SD)	38 (7.2)	38 (11.6)	
Diagnosis	None	PDA 61%	
		PD 39%	
		Comorbidity:MDD 13%	
*Meta-analysis*			
Participants, *N* (%)	245 (55%)	220 (45%)	465
Male, *N* (%)	76	88	164 (34%)
Female, *N* (%)	169	132	301 (66%)
Age, years (SD)	38 (7.3)	38 (11)	
Diagnosis	None	PDA 65%	
		PD 35%	
		Comorbidity:MDD 13%	
*Dex-treatment study*			
Participants, *N* (%)	29 (41%)	42 (59%)	71
Male, *N* (%)	0	0	0
Female, *N* (%)	29	42	71
Age, years (SD)	44 (11.4)	44 (13.7)	
Diagnosis	None	MDD	

*PDA* panic disorder with agoraphobia, *PD* panic disorder without agoraphobia, *MDD* major depressive disorder

Control subjects were recruited from a Munich-based community sample and screened for the absence of axis I psychiatric disorders using the Munich version of the Composite International Diagnostic Interview^32^. Controls were age-matched and sex-matched with patients.

To reduce confounding due to effects of drug treatment, both patients and controls were free of psychotropic medication for at least 4 weeks before the blood draw. All subjects were Caucasian and provided written informed consent. The Ethics Committee of the Ludwig Maximilians University, Munich, Germany, in accordance with the Declaration of Helsinki approved all procedures.

### Microarray processing and quality control in the MPIP PD cohort

Genomic DNA was extracted from peripheral blood using the Gentra Puregene Blood Kit (Qiagen). DNA quality and quantity was assessed using NanoDrop 2000 Spectrophotometer (Thermo Scientific) and Quant-iT Picogreen (Invitrogen). To minimize batch effects, samples were randomized with respect to case-control status, sex and age.

Genomic DNA was bisulfite converted using the Zymo EZ-96 DNA Methylation Kit (Zymo Research) and DNA methylation levels were assessed for >480,000 CpG sites using the Illumina HumanMethylation450 BeadChip array. Hybridization and processing were performed according to the instructions of the manufacturer.

The Bioconductor R package *minfi* (version 1.10.2) was used for the quality control of methylation data including intensity read outs, normalization, cell type composition estimation, *β*-value and *M*-value calculation. Outliers, i.e., samples whose behavior deviated from that of others in terms of median intensity, were excluded from the analysis (*N* = 3 in the discovery sample, *N* = 5 in the replication sample) as well as samples with a discordant methylation-predicted vs. reported sex (*N* = 1 in the replication sample).

Failed probes were excluded based on a detection *P*-value larger than 0.01 in >50% of the samples. X and Y chromosome were removed to avoid a possible gender effect and also non-specific binding probes^33^. We also excluded probes if single nucleotide polymorphisms (SNPs) were documented in the interval for which the Illumina probe is designed to hybridize. Probes located close (10 bp from query site) to a SNP which had a minor allele frequency of ≥0.05, as reported in the 1000 Genomes Project, were also removed. This yielded a total of around 425,000 CpG sites in the discovery and replication sample for further analysis.

The data were then normalized with functional normalization (FunNorm)^34^, an extension of quantile normalization included in the R package *minfi*.

Batch effects were identified by inspecting the association of principal components of the methylation levels with possible technical batches using linear regressions and visual inspection of principal component analysis plots using the Bioconductor R package *shinyMethyl* (version 0.99.3). Identified batch effects (i.e., bisulfite conversion plate and plate position) were removed using the Empirical Bayes’ method *ComBat* ^35^. Batch corrected *M*-values after *ComBat* were used for all further statistical analyses.

### Epigenome-wide association analysis

Linear regression models were fit for each probe to test for a case vs. control difference within the R package MatrixEQTL (version 2.1.1)^36^. Sex, age and imputed white blood cell distribution from the Houseman projection^37^ were included as covariates. Population stratification was investigated using multidimensional scaling and could not be observed (Supplementary Fig. S1). Significance after multiple testing was adjusted using false discovery rate (FDR) of 5%. As a first step all the samples were analyzed together (Table 1) but, given the higher prevalence of PD in females, we performed a sex-stratified analysis, first in the discovery and then in the replication sample. A fixed-effect meta-analysis across both samples was performed in Plink v1.9^38^ following identification of hits in the individual analyses.

In order to investigate whether we can find clusters of association in the epigenome-wide analysis, we performed the differentially methylated region (DMR) analysis on the combined results from both samples based on the input of individual *P*-values of at least 5e-05 and within 500 bp using Comb-P^39^.

### Targeted gene analysis

A high number of studies showed mostly single SNP associations in different genes with PD, however, the replicability of these findings was low. Therefore, we used three lines of approaches to select candidate genes for the targeted methylome analysis: (1) candidate genes from human genetic studies confirmed in the recent meta-analysis of different international PD cohorts (*TMEM132D*, *COMT*, *NPSR1* and *HTR2A*)^7^, (2) and/or having additional evidence from translational studies for anxiety and stress related-phenotypes (*CRH*, *CRHR1*, *ADCYAP1*, *ADCYAP1R1*, *FKBP5*, *SGK1*, *BDNF*, *HTR1A*)^8, 40–46^ and lastly, (3) genes containing loci with previous evidence for differential methylation in PD and anxiety disorders (*GAD1*, *OXTR*)^20, 47^. All the genes examined (*N* = 15) showed previous evidence of association with stress-related phenotypes not only in clinical (human) studies but also in preclinical (animal) studies^5,26,27, 48–52^.

The CpGs lying within the target genes were selected from the meta-analysis results of the epigenome-wide association study (EWAS) and FDR correction of 5% was applied for the number of CpGs included in the gene.

### Disease association analysis

To investigate a possible enrichment for specific pathways, we conducted a disease association analysis using Web Gestalt^53, 54^, DAVID^55, 56^ and the R-package DOSE^57^.

Tested genes for a disease enrichment were annotated from CpG sites with *P*-value < 0.001 in the meta-analysis results of the cases vs. controls EWAS in the whole sample (*N*_genes_ = 312), in the female subset (*N*_genes_ = 428) and in the male subset (*N*_genes_ = 379). The analysis was background corrected for the Illumina HumanMethylation450 BeadChip array annotated genes.

### DNA Methylation age calculation

DNA methylation age was calculated from peripheral blood of patients and controls included in the discovery (*N* = 165) and replication sample (*N* = 300). DNA methylation-based age prediction was performed using the R code and statistical pipeline developed by Horvath^58^. This predictor was developed using 82 Illumina DNA methylation array datasets (*n* = 7,844) involving 51 healthy tissues and cell types^58^. The raw data were normalized using BMIQ normalization method^59^ implemented in the Horvath DNA methylation-based age predictor R script^58^. We then tested whether epigenetic age acceleration (∆-age), calculated by subtracting the actual chronological age from DNA methylation age^58^, was associated with case-control status. Since DNA methylation age is calculated from raw beta values, technical batches identified for discovery and replication sample (96-well plate) were included as covariates in the linear regression model together with age, sex and cell counts (Houseman and Horvath cell counts, specifically: PlasmaBlast, CD8pCD28nCD45Ran, CD8.naive, CD4T, NK, Mono, Gran).

### MPIP dexamethasone treatment study

Glucocorticoid-induced methylation and gene expression changes were examined in an independent sample of 71 Caucasian female subjects (29 healthy probands and 42 depressed) recruited at the MPIP. Recruitment strategies and characterization of participants have been previously described^60^. Baseline whole blood samples were obtained at 6 pm after 2 h of fasting and abstention from coffee and physical activity (baseline). Subjects then received 1.5 mg oral dexamethasone (DEX) and a second blood draw was performed at 9 pm 3 h after DEX ingestion (post-DEX). The study was approved by the local ethics committee and all individuals gave written informed consent.

### DNA methylation and gene expression arrays in the MPIP dexamethasone treatment study

Genomic DNA was extracted from whole blood using the Gentra Puregene Blood Kit (QIAGEN) and processed as for the MPIP PD cohort. DNA methylation levels were assessed for >480,000 CpG sites using the Illumina HumanMethylation450 BeadChip arrays. Whole blood RNA was collected using PAXgene Blood RNA Tubes (PreAnalytiX), processed as described previously^60^. Blood RNA was hybridized to Illumina HumanHT-12 v3 and v4 Expression BeadChips arrays. All methylation and gene expression array probes have been subjected to an extensive quality control including filtering by low p-detection value, normalization (FunNorm for methylation and VSN for gene expression data) and batch correction with ComBat as previously described in ref. ^61^. Cellular composition was estimated by using CellCode^62^.

### Statistical analysis in the MPIP dexamethasone treatment study

Methylation levels of cg07308824 were tested for association with gene expression levels of the *HECA* mRNA (ILMN_1770667) using a linear mixed effects model within the lme4 package^63^.

## Results

### Genome-wide methylation differences in discovery and replication samples

**Fig. 1:**
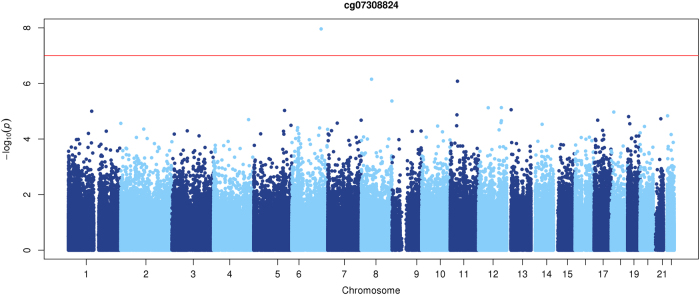
Manhattan plot of the Panic Disorder EWAS in females (meta-analysis results): the *x*-axis shows chromosomal position and the *y*-axis shows −log10 (P) The red line represents the multiple test threshold (*P* < 1.09 × 10^−7^)

**Fig. 2:**
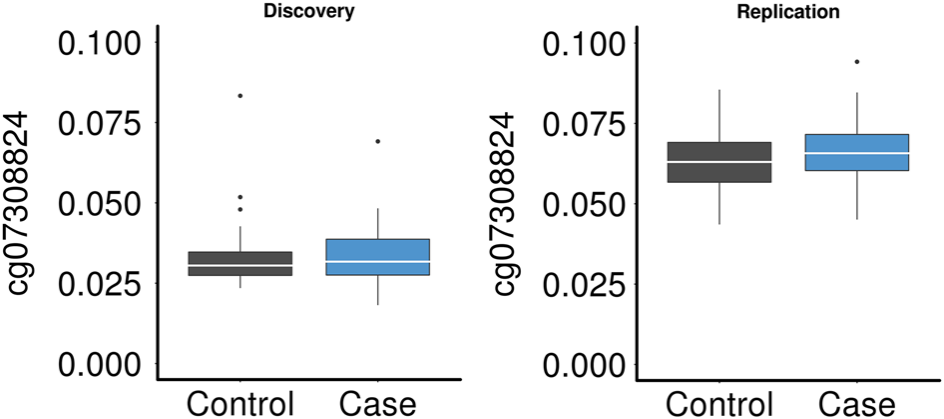
Box plot of DNA methylation levels for the genome-wide significant CpG in the discovery (P-adj = 0.046) and replication sample (*P* = 0.035)

Genome-wide associations were performed in the discovery sample, combined as well as stratified by gender. While no association survived correction for multiple testing in the overall samples and the male subset, one genome-wide association, cg07308824, surviving FDR of 5% (*P* = 1.094 × 10-7, P-adj = 0.046) was observed in the female-only discovery sample. QQ plots for each of the analyses are presented in the Supplementary Figs. S2–S3. cg07308824 is located in the promoter of the *HECA* gene and was hypermethylated in female PD patients (*N* = 49) compared to controls (*N* = 48). The association and the direction of the association could be replicated in the second sample (*P* = 0.035) and yielded a combined *P*-value of 1.651e-08 in the meta-analysis, that would again survive correction for multiple testing (P-adj = 0.004) (Figs. 1, 2).

The DMR analysis using Comb-P revealed no significantly associated regions.

### Targeted gene analysis

The targeted gene analysis (see Supplementary Tables S1–S15 for the complete list of genes tested) using the meta-analysis results, yielded in females one significant CpG each (surviving 5% FDR correction over the CpGs in the gene) in *ADCYAP1* (P-adj = 0.010) and *HTR1A* (P-adj = 0.041) (Supplementary Fig. S4). The same analysis yielded one significant CpG in *SGK1* (P-adj = 0.035) (Supplementary Fig. S5) in the whole sample and in males in fragile histidine triad (*FHIT*, P-adj = 0.010) and two significant CpGs in *HTR2A* (P-adj = 0.015 and P-adj = 0.029) (Supplementary Fig. S6) (Table 2). Single nominal associations have been found in the genes *ADCY1P1R1*, *BDNF*, *COMT*, *CRH*, *CRHR1*, *GAD1*, *OXTR* and *TMEM132D*. No differential methylation was detected for *NPSR1* between cases and controls.

**Table 2:** Targeted gene analysis results for the significant CpGs

Sample	Gene	CpG	P-adj
*Meta-analysis*			
Whole	*SGK1*	cg00959636	0.035
Males	*FHIT*	cg07351758	0.010
	*HTR2A*	cg09361691	0.015
		cg06476131	0.029
Females	*ADCYAP1*(PACAP)	cg13940693	0.010
	*HTR1A*	cg16280141	0.041

### Disease association analysis

An enrichment for psychiatric disorders could be found in the whole sample (bipolar disorder, *P* = 1.9e-2; mental disorders, *P* = 2.5e-2) and in females (response to antipsychotic treatment, *P* = 5.3e-2; ADHD, *P* = 9.5e-2) using DAVID^55, 56^.

### Functional characterization of significant results

**Fig. 3:**
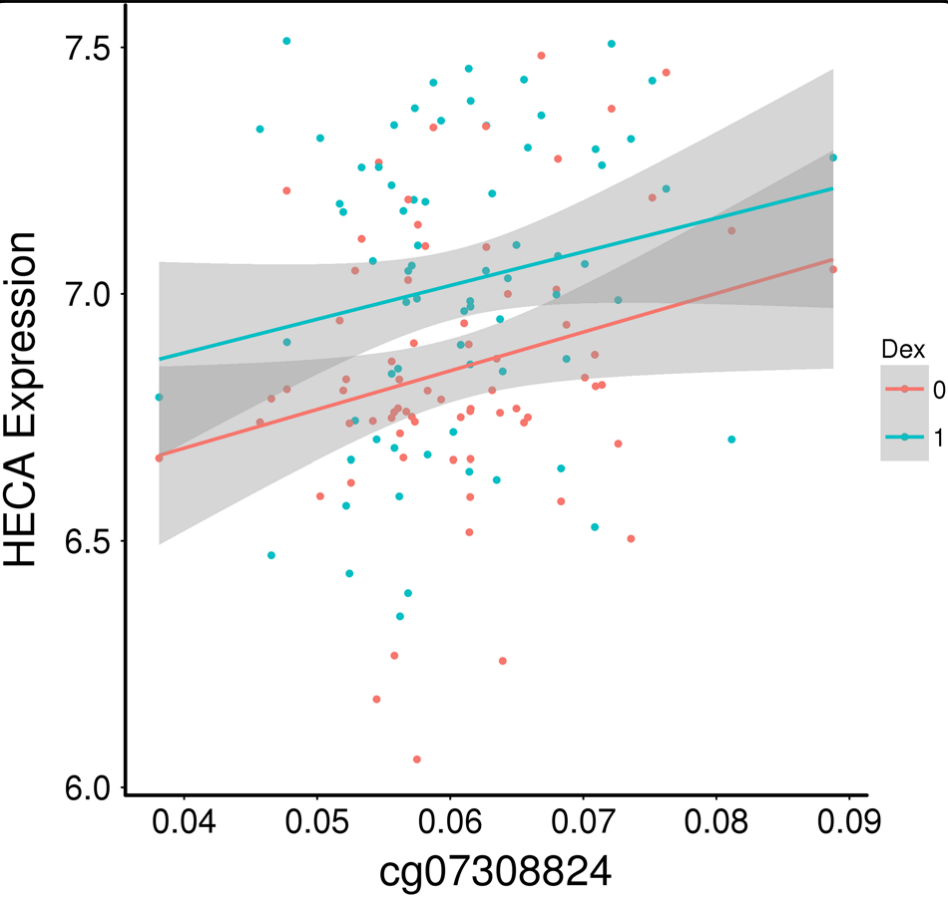
Scatterplot showing the association between DNA methylation (*x*-axis, beta values) and gene-expression (*y*-axis, VSN normalized array probe intensity) in an independent female sample at baseline (*P* = 0.046) and after induction by dexamethasone (*P* = 0.029)

To assess the functionality of the significant CpG methylation site, association of methylation levels with gene expression of the *HECA* mRNA was tested. Methylation at this CpG site was associated with mRNA expression of *HECA* (ILMN_1770667) both at baseline (*P* = 0.046) and after induction by dexamethasone (*P* = 0.029) (Fig. 3). Gene expression was significantly altered in the sample following dexamethasone induction (*P* = 8.78e-05) but not DNA methylation (*P* = 0.796), indicating that the significant association between gene expression and DNA methylation is specific and not due to dexamethasone.

### DNA Methylation age and case-control status

**Fig. 4:**
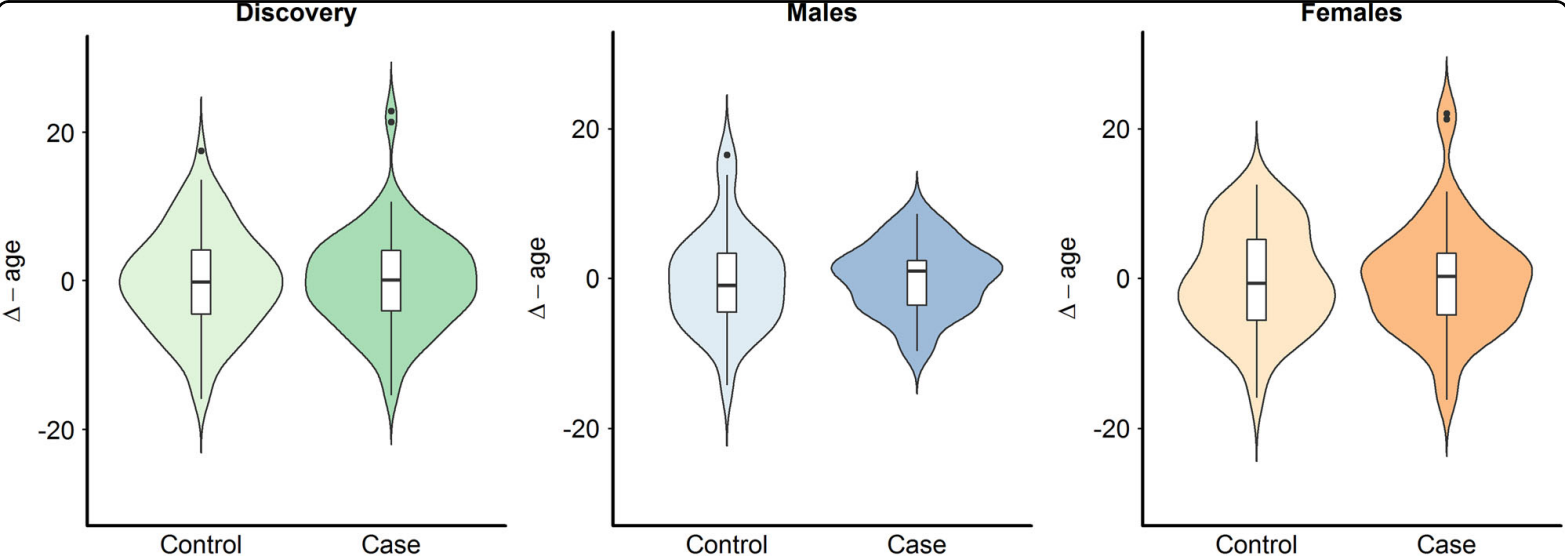
Violin plots of ∆-age by case-control status in the discovery sample From the left: whole sample (*P* = 0.980), males only (*P* = 0.835), and females only (*P* = 0.964)

PD is a strong stressor for the people affected and so far no studies have been carried out to determine whether patients affected by PD also develop age acceleration. To answer this question, we compared the ∆-age of PD patients with healthy controls in the whole discovery (*N* = 165, *P* = 0.980) and replication sample (*N* = 300, *P* = 0.282) and found no significant differences. We then stratified for gender and found no significant results in males (discovery: *N* = 68, *P* = 0.835; replication: *N* = 95, *P* = 0.467) as well as in females (discovery: *N* = 97, *P* = 0.964; replication: *N* = 204, *P* = 0.402) (Fig. 4, Supplementary Fig. S7).

## Discussion

Genome-wide association of whole blood DNA methylation with PD cases and matched controls identified a locus (cg07308824), which was hypermethylated in female PD patients compared to healthy controls. This locus was also associated with case-control status in females in another independent sample and results were further confirmed with a meta-analysis (*N* = 301). No methylation differences were identified at genome-wide level taking both genders together. This is the first and biggest EWAS for PD in a population with European background.

**Fig. 5:**
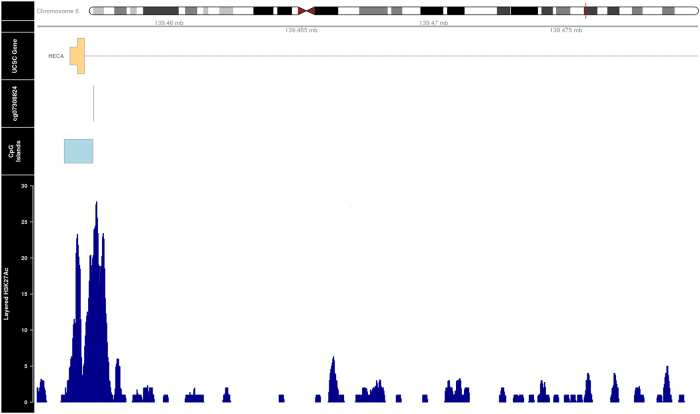
Annotation of the genome-wide significant CpG located in the *HECA* gene The top panel contains the *HECA* gene model, located on Chr 6. The other two panels show the genome-wide significant CpG and the CpG island where the CpG is located. The bottom panel shows the levels of enrichment of the H3K27Ac mark in the *HECA* gene. Data were obtained from UCSC Genome Browser and plotted using the R package Gviz^87^

The methylation locus that we identified in females is located in the intragenic and enhancer region^64^ of the Homo sapiens headcase homolog (Drosophila) (*HECA*) gene on Chromosome 6. The *HECA* gene is a cell cycle regulator and may play an important role in human cancers, e.g., hepatocellular carcinoma^65^; however, only a few publications about this gene are available to date. The potential functional relevance of cg07308824 was further investigated in the UCSC Genome Browser^64^. An overlap was observed between the location of cg07308824 probe and histone 3 lysine 27 acetylation (H3K27Ac) on seven cell lines from ENCODE^66^ (Fig. 5). *H3K27Ac* was previously found near to active regulatory elements suggesting that the sequence where the probe is located is functional^67^. The *HECA* gene is expressed in brain at lower levels compared to blood (Supplementary Fig. S8) but we could not see any correlation between the methylation levels of the significant CpG found in blood and brain, which could indicate that the relevance of these results might be limited to blood. No significant correlations were found between cg07308824 methylation levels and four different brain regions (i.e., prefrontal cortex, superior temporal gyrus, entorhinal cortex and cerebellum) in a linear regression model using a publicly available data set^16^(Supplementary Fig. S9).

It is noteworthy considering that methylation levels of the identified locus showed a significant correlation with gene expression levels of the *HECA* gene in another independent female sample, which points to the functional relevance of the observed methylation change. The fact that the direction of the association is positive (higher gene expression correlated with higher methylation) may be explained by the intragenic location of the significant locus. It has indeed previously been shown^68^ that a positive correlation with gene expression is expected for CpG probes located in the body of the gene and a negative correlation is expected for CpG probes located close to a gene’s TSS. The authors also report that however this is only partially verified, with one-third of the latter type showing a positive correlation and nearly half of the former type showing a negative correlation.

Notably, the significant changes in DNA methylation presented here are small with less than 1% difference (0.08%) but replicable. While the functional relevance of such small changes is debatable, these effect sizes are in line with other EWAS in psychiatry, including the recent small Japanese EWAS in PD^22^, where 40 significant CpGs with overall low methylation (mostly under 0.05%) differences have been detected. Sex-specific associations were not reported, most likely because of the small sample size. Furthermore, similar effect sizes were observed in other methylome studies of psychiatric disorders (schizophrenia^24^) and other complex diseases (rheumatoid arthritis^69^, multiple sclerosis^70^ and Alzheimer’s disease^71^). The EWAS in schizophrenia is largest EWAS in the psychiatric field to date, with 689 schizophrenia patients and 645 controls included in the analysis and shows methylation differences lower than 0.02% that replicate in an independent cohort. More work needs to be performed to understand what contributes to these small differences (e.g., slight differences in cell composition, genotype differences or differences in environmental exposures) and if they are informative beyond serving as biomarker.

Female-specific DNA methylation changes in PD have been previously shown both in mice^72^ and in humans. A female-specific association and/or correlation of negative life events with decreased overall methylation levels has been shown for *GAD1* and *MAOA* ^20, 73^. In contrast, female-specific effects in terms of increased methylation levels of promoter region were observed in the *FOXP3* gene for PD^18^. Sex-specific findings regarding the methylation pattern have been also detected in depression, which is highly comorbid with PD, and psychosis^74, 75^.

Interestingly, the disease association analysis shows an enrichment for psychiatric disorders in the whole sample and in females, but not in males.

In our analysis with Comb-P, no evidence for clusters of differential methylation per gene could be detected, indicating that associated CpGs do not have adjacent signals. The genome-wide findings are thus not supported by DMR analysis. However, the structure of the 450k Illumina array has many loci that are only interrogated with single CpGs, especially in enhancer regions, so that single significant CpGs could reflect a differentially methylated region, but since no adjacent CpGs are probed, this cannot be assessed using only array data. Here bisulfite sequencing approaches are needed.

One of the limitation of the study is that we could not correct for the smoking status of the subjects, due to lack of information. We verified though that our significant genome-wide hit was not one of the top-associated CpGs in the biggest EWAS for cigarette smoking^76^. Another limitation is the lower number of males compared to females, which is due to the higher prevalence of the disease in the latter. This might also explain why no significant results were found in the male subset but only in females. For this reason a bigger study with a higher number of subjects, with possibly the same ratio between genders, is necessary in order to confirm the sex-specificity of our findings.

We have to point out that we observe increased lambdas in the QQ plots of the replication sample despite correction for population stratification and methodological issues, such as DNA extraction and batch effects. It is noteworthy that female subjects of the replication sample seem to contribute to the higher inflation in our study. Other previously published EWAS in psychiatric disorders have shown similar or even higher inflation^24,25, 77–79^. The different sample size of discovery and replication sample as well as between female and male subjects might be one reason for these effects. The sample size of female subjects is doubled in the replication sample (97 vs. 204). If we extrapolate the inflation factor from discovery to replication sample^80^, we see an increase of lambda to 1.11. In our study, we saw the association in the *HECA* gene first in the discovery sample which showed only moderate inflation and could then confirm the finding in the second sample and meta-analysis, suggesting that this finding cannot be attributed just to the inflation effects in the replication sample. Additionally, if we correct for population stratification using the inflation factor qualitatively results are not altered.

A targeted gene approach was subsequently applied, with the aim of investigating if genes previously associated with PD or anxiety-related phenotypes are affected at the methylation level. Five of the genes we analyzed, i.e., *HTR1A*, *HTR2A*, *ADCYAP1* (pituitary adenylate cyclase-activating polypeptide (PACAP)), *FHIT* and *SGK1* showed different methylation patterns in PD patients compared to controls. For these genes, the evidence for a correlation with anxiety disorders at the genetic level could be confirmed in our data at the epigenetic level. SGK1 (serum/glucocorticoid regulated kinase 1) is one of the key player in the mediation of fast and chronic stress response and, therefore, could be implicated in the transition of the environmental stress influences via methylation^43^. It seems to play a role in the expression of conditioned fear in the animal model^27^ and is one target of miRNAs in the glucocorticoid pathway affecting neurogenesis and leading to anxiogenic and depressiogenic behavior in mice^81^. Additional evidence from human studies point to the implication of this gene in the pathophysiology of traumatic stress, e.g., PTSD^82^ and our results point to a possible involvement in PD as well. The second gene which is implicated in the stress response regulation is *PACAP*. Ressler et al. could demonstrate a female-specific significant correlation of the PACAP38 peptide concentration in blood with PTSD symptoms and diagnosis^83^. In line with these previous findings, we could also demonstrate a female-specific significant methylation difference in one locus between cases and controls, suggesting that this gene may play an important role in long-lasting stress dependent pathophysiology in PD or other anxiety disorders. Similarly, a female-specific methylation difference could be shown for the gene *HTR1A*. This serotonin receptor is the most abundant of all serotonin receptors in the brain and *HTR1A* variants have been shown to be associated with depression and defensive behavior in PD patients^41^. So far, there is no evidence for gender-specific implication of *HTR1A* gene in PD or other mental disorders. There are instead already previous studies showing an association of *HTR2A* and PD^7, 84^, and supporting evidence comes from our results in males. Similarly, variants in the gene *FHIT* (fragile histidine triad) were nominally associated in GWAS studies with anxiety^85^ and PD^5^, and were not gender-specific. In a recent huge meta-analysis for broad depression phenotype, several variants in *FHIT* were among the most significant hits^86^. However, there was no difference in the burden of depressive symptoms or depression diagnosis between the male and female group in our study, therefore, we cannot refer our finding to depression as bias. For *HTR1A*, *HTR2A* and *FHIT*, gender-specific effects presented here need to be replicated and elucidated in further studies.

In the targeted gene analysis we observe five significant results, which is more than expected by chance (2–3 expected by chance). We corrected gene-wide because each gene was regarded as separate analysis driven by the positive evidence for association with anxiety phenotypes. The results might be biased by the selection of the candidate gene itself as one limitation of the gene-targeted approach. Therefore, these findings, while supporting previous results, have to be taken with caution and replicated elsewhere.

Another aim of the study was to determine the effect of PD on epigenetic aging, as measured with the epigenetic clock^58^. We found no differences between PD patients and healthy controls in the discovery and replication sample, also when separated by gender. This might be explained by the heterogeneity of the samples in terms of age. It was showed by Zannas et al.^61^ that the effect of personal life stress on ∆-age is stronger in older as compared to younger people. This suggests that the effects in our study, if presents, might be diluted by the age-range. Additionally, the effect sizes of PD on epigenetic age might be low and higher sample sizes are needed to detect significant changes. As cumulative lifetime stress has been shown to contribute to faster epigenetic aging^61^, analysis of other phenotypes, such as number of panic attacks or duration of the disorder, could be more effective in uncovering epigenetic aging effects of PD. In summary, our study examines epigenome-wide differences in peripheral blood for PD patients. Our results point to possible sex-specific methylation changes in the *HECA* gene for PD but overall highlight that this disorder is not associated with extensive changes in DNA methylation pattern in peripheral blood.

## Electronic supplementary material

Supplemental Material
