# Supplementary material for: “DNA Methylation signatures in panic disorder”
Source: Transl Psychiatry. 2017 Dec 18;7:1287. doi: 10.1038/s41398-017-0026-1 (PMC5802688; doi:10.1038/s41398-017-0026-1)
Supplement: Supplementary file 1 — Supplemental Material [file 41398_2017_26_MOESM1_ESM.docx]

**Table S1-S15: Targeted gene analysis**

List of all the CpGs tested for every target gene. Beta and P-values refer to the EWAS meta-analysis results in the whole sample and stratified by gender.

| ADCYAP1 (PACAP) | | | | | | | | | |
| --- | --- | --- | --- | --- | --- | --- | --- | --- | --- |
|  | **Whole** | | | **Males** | | | **Females** | | |
| Probe | **Beta** | **P-value** | **FDR (Gene)** | **Beta** | **P-value** | **FDR (Gene)** | **Beta** | **P-value** | **FDR (Gene)** |
| cg00067606 | -0.0504 | 0.4317 | 0.949447 | -0.2272 | 0.03499 | 0.28668 | 0.0449 | 0.5866 | 0.900692 |
| cg04105966 | -0.0066 | 0.878 | 0.949447 | -0.0781 | 0.3986 | 0.8712 | -0.0082 | 0.8722 | 0.9198 |
| cg06372303 | 0.0034 | 0.9629 | 0.9629 | -0.0466 | 0.7303 | 0.8712 | 0.1528 | 0.1048 | 0.4062 |
| cg07211875 | 0.0098 | 0.8622 | 0.949447 | 0.0276 | 0.7744 | 0.8712 | 0.0425 | 0.5534 | 0.900692 |
| cg07376535 | 0.045 | 0.496 | 0.949447 | -0.0348 | 0.7729 | 0.8712 | 0.1765 | 0.03384 | 0.24714 |
| cg07788286 | -0.0116 | 0.8967 | 0.949447 | -0.3214 | 0.06718 | 0.30231 | -0.028 | 0.798 | 0.9198 |
| cg10384245 | 0.045 | 0.3765 | 0.949447 | 0.0368 | 0.6656 | 0.8712 | 0.0847 | 0.2008 | 0.4062 |
| cg11402363 | 0.0815 | 0.2328 | 0.949447 | 0.0413 | 0.7456 | 0.8712 | 0.0261 | 0.7596 | 0.9198 |
| cg11771234 | 0.0213 | 0.7908 | 0.949447 | 0.2461 | 0.0894 | 0.32184 | -0.0479 | 0.6312 | 0.900692 |
| cg11859607 | -0.0491 | 0.352 | 0.949447 | -0.0317 | 0.7281 | 0.8712 | 0.0068 | 0.9198 | 0.9198 |
| cg13940693 | 0.0084 | 0.8805 | 0.949447 | -0.1812 | 0.03518 | 0.28668 | 0.2438 | 0.000593 | **0.010674** |
| cg14200170 | -0.127 | 0.03223 | 0.58014 | 0.0071 | 0.9451 | 0.9481 | -0.0347 | 0.6505 | 0.900692 |
| cg14479567 | 0.0301 | 0.7072 | 0.949447 | -0.0782 | 0.5644 | 0.8712 | 0.136 | 0.1837 | 0.4062 |
| cg14489474 | -0.0192 | 0.8281 | 0.949447 | -0.0642 | 0.6738 | 0.8712 | -0.2295 | 0.04119 | 0.24714 |
| cg14908653 | -0.0248 | 0.7178 | 0.949447 | -0.2592 | 0.04778 | 0.28668 | -0.0192 | 0.8178 | 0.9198 |
| cg17439660 | 0.0661 | 0.3505 | 0.949447 | 0.0834 | 0.4905 | 0.8712 | 0.1198 | 0.2031 | 0.4062 |
| cg21331088 | -0.0123 | 0.7441 | 0.949447 | 0.0675 | 0.2967 | 0.8712 | -0.0696 | 0.1557 | 0.4062 |
| cg22374233 | -0.0528 | 0.3309 | 0.949447 | -0.006 | 0.9481 | 0.9481 | 0.0956 | 0.1964 | 0.4062 |

| ADCYAP1R1 (PACAP receptor) | | | | | | | | | |
| --- | --- | --- | --- | --- | --- | --- | --- | --- | --- |
|  | **Whole** | | | **Males** | | | **Females** | | |
| Probe | **Beta** | **P-value** | **FDR (Gene)** | **Beta** | **P-value** | **FDR (Gene)** | **Beta** | **P-value** | **FDR (Gene)** |
| cg01556466 | -0.1996 | 0.03595 | 0.670383 | -0.1541 | 0.3582 | 0.618709 | -0.1724 | 0.1913 | 0.841067 |
| cg02418899 | -0.0239 | 0.7897 | 0.9983 | 0.0307 | 0.8359 | 0.9329 | 0.1 | 0.3876 | 0.841067 |
| cg02846790 | 0.0589 | 0.3935 | 0.830722 | -0.0105 | 0.9329 | 0.9329 | -0.0195 | 0.8211 | 0.976041 |
| cg03447880 | -0.0453 | 0.322 | 0.830722 | -0.0539 | 0.5013 | 0.793725 | -0.0366 | 0.5312 | 0.841067 |
| cg04879561 | -0.0016 | 0.9859 | 0.9983 | -0.1654 | 0.2648 | 0.54188 | 0.0909 | 0.4223 | 0.841067 |
| cg10000602 | -0.1618 | 0.1722 | 0.670383 | -0.4688 | 0.012 | 0.228 | -0.0014 | 0.9929 | 0.9951 |
| cg11218385 | -0.0547 | 0.3655 | 0.830722 | -0.0556 | 0.6069 | 0.887008 | -0.0797 | 0.2933 | 0.841067 |
| cg12140543 | 0.1192 | 0.2112 | 0.670383 | 0.0215 | 0.8924 | 0.9329 | 0.068 | 0.584 | 0.853538 |
| cg13886135 | 0.0369 | 0.693 | 0.9983 | -0.3401 | 0.04424 | 0.366637 | -0.089 | 0.4496 | 0.841067 |
| cg14785679 | 0.0227 | 0.7944 | 0.9983 | -0.2577 | 0.05789 | 0.366637 | -0.0187 | 0.8704 | 0.976041 |
| cg16621855 | 0.0237 | 0.6881 | 0.9983 | -0.0185 | 0.853 | 0.9329 | 0.012 | 0.8733 | 0.976041 |
| cg17822807 | 0.0309 | 0.6739 | 0.9983 | 0.2252 | 0.09804 | 0.406283 | 0.0645 | 0.4884 | 0.841067 |
| cg18421840 | 2.00E-04 | 0.9983 | 0.9983 | -0.1469 | 0.2852 | 0.54188 | 6.00E-04 | 0.9951 | 0.9951 |
| cg19317517 | -0.1125 | 0.1589 | 0.670383 | -0.2136 | 0.1283 | 0.406283 | 0.077 | 0.4528 | 0.841067 |
| cg21619594 | -0.0942 | 0.2117 | 0.670383 | -0.1646 | 0.1855 | 0.440563 | 0.1132 | 0.2545 | 0.841067 |
| cg21844005 | -0.0281 | 0.7024 | 0.9983 | 0.1969 | 0.1213 | 0.406283 | 0.0733 | 0.4511 | 0.841067 |
| cg22963629 | -0.0108 | 0.8824 | 0.9983 | -0.1733 | 0.1785 | 0.440563 | 0.0236 | 0.804 | 0.976041 |
| cg24384519 | -0.0066 | 0.9329 | 0.9983 | -0.0557 | 0.6671 | 0.90535 | 0.2632 | 0.0117 | 0.2223 |
| cg25195987 | 0.1254 | 0.1718 | 0.670383 | -0.0373 | 0.8174 | 0.9329 | 0.1264 | 0.2964 | 0.841067 |

| BDNF | | | | | | | | | |
| --- | --- | --- | --- | --- | --- | --- | --- | --- | --- |
|  | **Whole** | | | **Males** | | | **Females** | | |
| Probe | **Beta** | **P-value** | **FDR (Gene)** | **Beta** | **P-value** | **FDR (Gene)** | **Beta** | **P-value** | **FDR (Gene)** |
| cg00298481 | -0.1265 | 0.285 | 0.855536 | 0.1718 | 0.3485 | 0.7675 | 0.097 | 0.5367 | 0.934125 |
| cg01418645 | 0.078 | 0.3517 | 0.855536 | -0.0977 | 0.4971 | 0.771184 | 0.1157 | 0.2892 | 0.934125 |
| cg05189570 | 0.0183 | 0.8676 | 0.915208 | -0.107 | 0.573 | 0.771184 | 0.08 | 0.5722 | 0.934125 |
| cg06025631 | 0.0686 | 0.4779 | 0.855536 | 0.2301 | 0.1765 | 0.735417 | 0.0388 | 0.7473 | 0.934125 |
| cg06260077 | -0.0075 | 0.938 | 0.938 | 0.0983 | 0.5861 | 0.771184 | -0.0024 | 0.9835 | 0.9835 |
| cg06979684 | -0.0738 | 0.3139 | 0.855536 | -0.2008 | 0.1077 | 0.6975 | -0.1534 | 0.09701 | 0.934125 |
| cg07159484 | 0.0165 | 0.7782 | 0.915208 | 0.1106 | 0.2676 | 0.7675 | 0.1126 | 0.1304 | 0.934125 |
| cg07238832 | -0.0485 | 0.6191 | 0.915208 | -0.2511 | 0.134 | 0.6975 | 0.0158 | 0.8999 | 0.940833 |
| cg08388004 | 0.1013 | 0.2613 | 0.855536 | -0.1435 | 0.3347 | 0.7675 | 0.1149 | 0.3399 | 0.934125 |
| cg09492354 | -0.0477 | 0.6261 | 0.915208 | 0.0184 | 0.9114 | 0.96625 | -0.1694 | 0.1906 | 0.934125 |
| cg10558494 | -0.0967 | 0.2255 | 0.855536 | -0.2112 | 0.1395 | 0.6975 | 0.2338 | 0.01978 | 0.4945 |
| cg14291693 | -0.0895 | 0.3045 | 0.855536 | -0.0999 | 0.4771 | 0.771184 | 0.0543 | 0.6402 | 0.934125 |
| cg15014679 | -0.0583 | 0.4299 | 0.855536 | 0.0429 | 0.7265 | 0.864881 | -0.0516 | 0.5985 | 0.934125 |
| cg15313332 | 0.0617 | 0.4583 | 0.855536 | 0.0624 | 0.6666 | 0.83325 | 0.0605 | 0.5703 | 0.934125 |
| cg18117895 | -0.0904 | 0.2966 | 0.855536 | -0.1263 | 0.4298 | 0.7675 | -0.0523 | 0.6224 | 0.934125 |
| cg18354203 | -0.0276 | 0.6872 | 0.915208 | -0.1422 | 0.2245 | 0.7675 | -0.033 | 0.7032 | 0.934125 |
| cg18595174 | -0.0445 | 0.5585 | 0.915208 | -0.0171 | 0.8961 | 0.96625 | -0.0218 | 0.8232 | 0.940833 |
| cg20108357 | 0.0865 | 0.2285 | 0.855536 | -0.1808 | 0.1364 | 0.6975 | 0.0169 | 0.8554 | 0.940833 |
| cg20954537 | -0.0354 | 0.7109 | 0.915208 | 0.0945 | 0.5578 | 0.771184 | 0.1082 | 0.3856 | 0.934125 |
| cg23426002 | -0.0692 | 0.4791 | 0.855536 | -0.1361 | 0.4151 | 0.7675 | -0.0407 | 0.7343 | 0.934125 |
| cg23619332 | -0.0184 | 0.8786 | 0.915208 | -0.0191 | 0.9276 | 0.96625 | 0.0976 | 0.5288 | 0.934125 |
| cg23947039 | 0.1282 | 0.01451 | 0.36275 | 0.221 | 0.03315 | 0.6975 | 0.0266 | 0.6894 | 0.934125 |
| cg25962210 | 0.0639 | 0.4228 | 0.855536 | 0.1512 | 0.3036 | 0.7675 | 0.0548 | 0.5954 | 0.934125 |
| cg26057780 | -0.0133 | 0.864 | 0.915208 | -0.0024 | 0.987 | 0.987 | -0.0114 | 0.9032 | 0.940833 |
| cg27193031 | -0.0243 | 0.7932 | 0.915208 | -0.1441 | 0.3757 | 0.7675 | 0.0579 | 0.6272 | 0.934125 |

| COMT | | | | | | | | | |
| --- | --- | --- | --- | --- | --- | --- | --- | --- | --- |
|  | **Whole** | | | **Males** | | | **Females** | | |
| Probe | **Beta** | **P-value** | **FDR (Gene)** | **Beta** | **P-value** | **FDR (Gene)** | **Beta** | **P-value** | **FDR (Gene)** |
| cg00107488 | 0.0065 | 0.9401 | 0.9802 | 0.1968 | 0.1889 | 0.611578 | -0.1524 | 0.1805 | 0.736447 |
| cg00465975 | -0.0069 | 0.9244 | 0.9802 | 0.07 | 0.5822 | 0.860119 | -0.0169 | 0.8565 | 0.8834 |
| cg03205258 | -0.005 | 0.8893 | 0.9802 | -0.0318 | 0.5999 | 0.860119 | -0.0068 | 0.8834 | 0.8834 |
| cg03724721 | -0.0253 | 0.7949 | 0.9802 | 0.0681 | 0.7012 | 0.860119 | 0.0843 | 0.478 | 0.736447 |
| cg04856117 | -2.00E-04 | 0.9983 | 0.9983 | -0.1824 | 0.1728 | 0.611578 | 0.0153 | 0.8826 | 0.8834 |
| cg06045576 | 0.1157 | 0.204 | 0.935894 | 0.0784 | 0.6336 | 0.860119 | -0.2391 | 0.03039 | 0.464 |
| cg06346307 | 0.0518 | 0.5645 | 0.935894 | -0.4143 | 0.004372 | 0.126788 | 0.0222 | 0.8528 | 0.8834 |
| cg06787004 | 0.108 | 0.2796 | 0.935894 | 0.0729 | 0.6573 | 0.860119 | 0.1988 | 0.1327 | 0.736447 |
| cg06860277 | 0.0781 | 0.4868 | 0.935894 | 0.0689 | 0.7224 | 0.860119 | 0.0402 | 0.7786 | 0.8834 |
| cg07579946 | 0.0504 | 0.5809 | 0.935894 | -0.0743 | 0.6332 | 0.860119 | 0.1277 | 0.284 | 0.736447 |
| cg08289189 | -0.0089 | 0.8944 | 0.9802 | -0.0694 | 0.5459 | 0.860119 | 0.0445 | 0.6038 | 0.833813 |
| cg08730070 | 0.1632 | 0.04414 | 0.320015 | 0.2221 | 0.1532 | 0.611578 | 0.0999 | 0.3163 | 0.736447 |
| cg09926649 | -0.0831 | 0.2905 | 0.935894 | -0.169 | 0.1792 | 0.611578 | -0.0159 | 0.8779 | 0.8834 |
| cg10122187 | 0.0221 | 0.7038 | 0.971914 | -0.0258 | 0.7844 | 0.860119 | -0.0853 | 0.2765 | 0.736447 |
| cg10253022 | 0.0793 | 0.3768 | 0.935894 | -0.1159 | 0.4682 | 0.860119 | 0.0839 | 0.4529 | 0.736447 |
| cg11361387 | -0.0887 | 0.1439 | 0.83462 | -0.1254 | 0.1898 | 0.611578 | 0.0718 | 0.3931 | 0.736447 |
| cg12728623 | 0.0632 | 0.5121 | 0.935894 | 0.1581 | 0.3218 | 0.777683 | -0.0564 | 0.6594 | 0.833813 |
| cg13175282 | 0.2176 | 0.01142 | 0.320015 | 0.0379 | 0.8008 | 0.860119 | 0.1246 | 0.2558 | 0.736447 |
| cg16834011 | 0.1582 | 0.04337 | 0.320015 | 0.0384 | 0.7707 | 0.860119 | 0.2201 | 0.032 | 0.464 |
| cg18731680 | -0.0442 | 0.4924 | 0.935894 | 0.0352 | 0.7272 | 0.860119 | -0.0702 | 0.4219 | 0.736447 |
| cg18773129 | 0.0376 | 0.6234 | 0.951505 | 0.0064 | 0.9598 | 0.9598 | 0.0447 | 0.6613 | 0.833813 |
| cg19930203 | -0.162 | 0.0234 | 0.320015 | -0.0174 | 0.8799 | 0.911325 | 0.0444 | 0.6474 | 0.833813 |
| cg20709110 | -0.0033 | 0.9464 | 0.9802 | 0.0964 | 0.244 | 0.7076 | -0.0699 | 0.2569 | 0.736447 |
| cg21905167 | 0.0423 | 0.5738 | 0.935894 | -0.0626 | 0.6084 | 0.860119 | -0.1944 | 0.05099 | 0.492903 |
| cg21919834 | 0.0318 | 0.6771 | 0.971914 | -0.2054 | 0.1658 | 0.611578 | 0.1107 | 0.233 | 0.736447 |
| cg22546130 | -0.0231 | 0.7766 | 0.9802 | -0.3316 | 0.01465 | 0.212425 | 0.186 | 0.07032 | 0.50982 |
| cg23601416 | 0.0841 | 0.32 | 0.935894 | -0.147 | 0.3039 | 0.777683 | 0.0963 | 0.3826 | 0.736447 |
| cg25836061 | -0.0451 | 0.4905 | 0.935894 | -0.1444 | 0.1616 | 0.611578 | 0.0652 | 0.4825 | 0.736447 |
| cg27521571 | 0.0523 | 0.4265 | 0.935894 | -0.05 | 0.6345 | 0.860119 | 0.0763 | 0.3681 | 0.736447 |

| CRH | | | | | | | | | |
| --- | --- | --- | --- | --- | --- | --- | --- | --- | --- |
|  | **Whole** | | | **Males** | | | **Females** | | |
| Probe | **Beta** | **P-value** | **FDR (Gene)** | **Beta** | **P-value** | **FDR (Gene)** | **Beta** | **P-value** | **FDR (Gene)** |
| cg00603617 | 0.1035 | 0.2519 | 0.431829 | 0.0085 | 0.958 | 0.958 | -0.0175 | 0.8787 | 0.8787 |
| cg03405789 | -0.1706 | 0.02053 | 0.10688 | 0.0112 | 0.936 | 0.958 | -0.1043 | 0.2391 | 0.35865 |
| cg08215831 | 0.07 | 0.1503 | 0.3006 | -0.0141 | 0.8841 | 0.958 | 0.1449 | 0.01404 | 0.08424 |
| cg15971888 | -0.0269 | 0.7758 | 0.7758 | -0.1252 | 0.497 | 0.958 | -0.0546 | 0.6326 | 0.75912 |
| cg17305181 | -0.0466 | 0.3381 | 0.4508 | -0.0711 | 0.4154 | 0.958 | 0.0911 | 0.1394 | 0.248914 |
| cg18640030 | 0.1051 | 0.02672 | 0.10688 | 0.0258 | 0.7515 | 0.958 | 0.089 | 0.1452 | 0.248914 |
| cg19035496 | 0.0306 | 0.5702 | 0.68424 | 0.2023 | 0.04534 | 0.54408 | 0.0972 | 0.1404 | 0.248914 |
| cg20329958 | -0.1223 | 0.1017 | 0.24408 | -0.1603 | 0.1738 | 0.958 | -0.1859 | 0.05638 | 0.18723 |
| cg21240762 | 0.1022 | 0.3175 | 0.4508 | 0.1002 | 0.5859 | 0.958 | 0.029 | 0.8195 | 0.8787 |
| cg21878188 | 0.048 | 0.6343 | 0.691964 | -0.1515 | 0.3789 | 0.958 | 0.2398 | 0.06241 | 0.18723 |
| cg23027580 | 0.2001 | 0.009545 | 0.10688 | 0.0882 | 0.5185 | 0.958 | 0.2669 | 0.005015 | 0.06018 |
| cg23409074 | 0.0995 | 0.0688 | 0.2064 | -0.0484 | 0.6452 | 0.958 | 0.0608 | 0.3636 | 0.4848 |

| CRHR1 | | | | | | | | | |
| --- | --- | --- | --- | --- | --- | --- | --- | --- | --- |
|  | **Whole** | | | **Males** | | | **Females** | | |
| Probe | **Beta** | **P-value** | **FDR (Gene)** | **Beta** | **P-value** | **FDR (Gene)** | **Beta** | **P-value** | **FDR (Gene)** |
| cg00022871 | 0.0498 | 0.637 | 0.975 | -0.0833 | 0.63 | 0.987553 | 0.0666 | 0.624 | 0.763688 |
| cg00025823 | 0.0099 | 0.8965 | 0.975 | 0.0873 | 0.5061 | 0.987553 | 0.0659 | 0.5093 | 0.730414 |
| cg04856689 | -0.0952 | 0.1 | 0.823175 | -0.156 | 0.128 | 0.608 | -0.0457 | 0.5304 | 0.730414 |
| cg07778819 | -0.0903 | 0.1733 | 0.823175 | 0.0083 | 0.9416 | 0.9922 | 0.0538 | 0.5382 | 0.730414 |
| cg11338426 | -0.172 | 0.1522 | 0.823175 | -0.3406 | 0.1064 | 0.608 | -0.0979 | 0.506 | 0.730414 |
| cg11731737 | 0.0045 | 0.9309 | 0.975 | -0.0199 | 0.8275 | 0.987553 | 0.0164 | 0.8008 | 0.845289 |
| cg13521908 | -0.1154 | 0.2961 | 0.892156 | 0.0257 | 0.8836 | 0.987553 | -0.2714 | 0.0674 | 0.730414 |
| cg13947929 | -0.054 | 0.4226 | 0.892156 | -0.0012 | 0.9922 | 0.9922 | 0.0683 | 0.4239 | 0.730414 |
| cg15607306 | 0.066 | 0.4151 | 0.892156 | 0.0319 | 0.8166 | 0.987553 | 0.0015 | 0.9889 | 0.9889 |
| cg16642545 | 0.0608 | 0.4768 | 0.90592 | -0.1068 | 0.4444 | 0.987553 | -0.1061 | 0.3516 | 0.730414 |
| cg16830379 | -0.0824 | 0.3382 | 0.892156 | -0.2499 | 0.08074 | 0.608 | -0.0785 | 0.4758 | 0.730414 |
| cg18534039 | -0.0078 | 0.9323 | 0.975 | -0.0653 | 0.7171 | 0.987553 | 0.0503 | 0.6598 | 0.763688 |
| cg24063856 | -0.0368 | 0.5671 | 0.975 | -0.0778 | 0.5036 | 0.987553 | -0.0333 | 0.6833 | 0.763688 |
| cg24353392 | 0.003 | 0.975 | 0.975 | -0.1281 | 0.4467 | 0.987553 | 0.1821 | 0.1323 | 0.730414 |
| cg24394631 | 0.0156 | 0.7464 | 0.975 | -0.0667 | 0.4246 | 0.987553 | 0.0592 | 0.3558 | 0.730414 |
| cg26656751 | -0.0145 | 0.7419 | 0.975 | -0.1694 | 0.0119 | 0.2261 | -0.0373 | 0.5311 | 0.730414 |
| cg27410679 | 0.0867 | 0.3052 | 0.892156 | 0.0731 | 0.5889 | 0.987553 | -0.0864 | 0.4364 | 0.730414 |
| cg27503360 | -0.1991 | 0.05718 | 0.823175 | -0.0279 | 0.8809 | 0.987553 | -0.18 | 0.1794 | 0.730414 |
| cg27551605 | -0.0097 | 0.8815 | 0.975 | 0.0193 | 0.8578 | 0.987553 | 0.1369 | 0.1014 | 0.730414 |

| FHIT | | | | | | | | | |
| --- | --- | --- | --- | --- | --- | --- | --- | --- | --- |
|  | **Whole** | | | **Males** | | | **Females** | | |
| Probe | **Beta** | **P-value** | **FDR (Gene)** | **Beta** | **P-value** | **FDR (Gene)** | **Beta** | **P-value** | **FDR (Gene)** |
| cg00071984 | 0.1004 | 0.174 | 0.72501 | -0.02 | 0.8903 | 0.9912 | 0.094 | 0.2899 | 0.9447 |
| cg00506250 | -0.0727 | 0.5265 | 0.982695 | -0.1749 | 0.3757 | 0.9912 | -0.003 | 0.9838 | 0.9951 |
| cg00658590 | 0.1067 | 0.2702 | 0.820393 | 0.0401 | 0.8001 | 0.9912 | 0.0962 | 0.436 | 0.989733 |
| cg00721771 | 0.0645 | 0.3802 | 0.982695 | 0.0014 | 0.9912 | 0.9912 | 0.1003 | 0.31 | 0.9447 |
| cg01556706 | 0.0182 | 0.7415 | 0.982695 | -0.146 | 0.1233 | 0.80145 | -0.0193 | 0.7822 | 0.9951 |
| cg02923224 | 0.0343 | 0.6361 | 0.982695 | -0.0808 | 0.5541 | 0.9912 | 0.046 | 0.613 | 0.9951 |
| cg03060986 | 0.2573 | 0.002328 | 0.090792 | -0.1763 | 0.2237 | 0.9912 | 0.1497 | 0.1831 | 0.826367 |
| cg03319184 | -0.0027 | 0.9677 | 0.9892 | 0.0017 | 0.9877 | 0.9912 | -0.0384 | 0.6607 | 0.9951 |
| cg03610148 | 0.1937 | 0.05035 | 0.63102 | -0.0018 | 0.991 | 0.9912 | 0.2637 | 0.04792 | 0.6357 |
| cg04216480 | 0.0827 | 0.2945 | 0.820393 | -0.026 | 0.8453 | 0.9912 | 0.1799 | 0.08276 | 0.6357 |
| cg04383442 | 0.1798 | 0.05617 | 0.63102 | -0.148 | 0.3314 | 0.9912 | 0.0331 | 0.7881 | 0.9951 |
| cg04835638 | 0.1208 | 0.1859 | 0.72501 | 0.0535 | 0.7414 | 0.9912 | 0.2417 | 0.0362 | 0.6357 |
| cg05645292 | 0.1099 | 0.1488 | 0.72501 | 0.0926 | 0.4818 | 0.9912 | -0.0221 | 0.8179 | 0.9951 |
| cg05709770 | -0.1124 | 0.1202 | 0.72501 | -0.2657 | 0.03914 | 0.305292 | 0.01 | 0.9124 | 0.9951 |
| cg07351758 | -0.0112 | 0.9048 | 0.982695 | -0.6192 | 0.000274 | **0.010694** | 0.1809 | 0.1136 | 0.6357 |
| cg08223225 | -0.0015 | 0.9892 | 0.9892 | -0.0515 | 0.8039 | 0.9912 | 0.2412 | 0.07833 | 0.6357 |
| cg10763247 | 0.1066 | 0.2341 | 0.820393 | -0.1009 | 0.4984 | 0.9912 | 0.0111 | 0.9248 | 0.9951 |
| cg11815980 | -0.0256 | 0.7883 | 0.982695 | -0.0703 | 0.6783 | 0.9912 | 0.0922 | 0.4568 | 0.989733 |
| cg13679804 | 0.0153 | 0.7701 | 0.982695 | 0.0627 | 0.4471 | 0.9912 | 0.0545 | 0.456 | 0.989733 |
| cg13745692 | 0.0543 | 0.5126 | 0.982695 | 0.0468 | 0.7276 | 0.9912 | 0.0724 | 0.5194 | 0.9951 |
| cg14147855 | -0.0305 | 0.7321 | 0.982695 | -0.0088 | 0.9542 | 0.9912 | 7.00E-04 | 0.9951 | 0.9951 |
| cg15135842 | 0.0198 | 0.8306 | 0.982695 | -0.1549 | 0.3627 | 0.9912 | -0.0535 | 0.6371 | 0.9951 |
| cg15238012 | -0.0655 | 0.4165 | 0.982695 | -0.1304 | 0.3482 | 0.9912 | 0.0924 | 0.3613 | 0.989733 |
| cg15970800 | 0.0303 | 0.7785 | 0.982695 | 0.0448 | 0.8196 | 0.9912 | 0.02 | 0.8825 | 0.9951 |
| cg16806041 | -0.0484 | 0.5667 | 0.982695 | -0.0395 | 0.7892 | 0.9912 | 0.0181 | 0.8687 | 0.9951 |
| cg17087356 | 0.1739 | 0.06472 | 0.63102 | 0.1753 | 0.2861 | 0.9912 | 0.0325 | 0.7865 | 0.9951 |
| cg17894779 | 0.0977 | 0.2918 | 0.820393 | -0.0815 | 0.6151 | 0.9912 | 0.1838 | 0.1141 | 0.6357 |
| cg19282443 | 0.1679 | 0.1161 | 0.72501 | 0.0901 | 0.6283 | 0.9912 | -0.0136 | 0.92 | 0.9951 |
| cg19729536 | -0.0066 | 0.9323 | 0.982695 | -0.0545 | 0.6815 | 0.9912 | -0.0207 | 0.8386 | 0.9951 |
| cg20366397 | 0.0305 | 0.7811 | 0.982695 | -0.1105 | 0.5522 | 0.9912 | 0.0604 | 0.6695 | 0.9951 |
| cg20517149 | -0.0577 | 0.4933 | 0.982695 | -0.3129 | 0.02887 | 0.305292 | -0.0653 | 0.5301 | 0.9951 |
| cg22380007 | 0.0081 | 0.8393 | 0.982695 | -0.2264 | 0.00176 | 0.03432 | 0.0521 | 0.2877 | 0.9447 |
| cg22533480 | -0.0134 | 0.8636 | 0.982695 | 0.0472 | 0.7022 | 0.9912 | -0.181 | 0.07068 | 0.6357 |
| cg23222057 | -0.0064 | 0.9299 | 0.982695 | -0.2589 | 0.03471 | 0.305292 | -0.0534 | 0.5666 | 0.9951 |
| cg23737061 | -0.0633 | 0.4357 | 0.982695 | -0.09 | 0.4774 | 0.9912 | -0.0899 | 0.4052 | 0.989733 |
| cg25724751 | 0.0331 | 0.6593 | 0.982695 | -0.1619 | 0.1836 | 0.9912 | -0.0011 | 0.9913 | 0.9951 |
| cg25921543 | 0.044 | 0.6272 | 0.982695 | 0.0909 | 0.5333 | 0.9912 | 0.1528 | 0.1907 | 0.826367 |
| cg26358659 | 0.0211 | 0.8371 | 0.982695 | -0.2083 | 0.247 | 0.9912 | 0.0218 | 0.8667 | 0.9951 |
| cg27254860 | 0.105 | 0.1714 | 0.72501 | 0.0114 | 0.933 | 0.9912 | 0.0976 | 0.3149 | 0.9447 |

| FKBP5 | | | | | | | | | |
| --- | --- | --- | --- | --- | --- | --- | --- | --- | --- |
|  | **Whole** | | | **Males** | | | **Females** | | |
| Probe | **Beta** | **P-value** | **FDR (Gene)** | **Beta** | **P-value** | **FDR (Gene)** | **Beta** | **P-value** | **FDR (Gene)** |
| cg00140191 | -0.0095 | 0.9156 | 0.9156 | -0.076 | 0.6362 | 0.956114 | 0.0457 | 0.682 | 0.909333 |
| cg00862770 | -0.0144 | 0.875 | 0.9156 | -0.2893 | 0.05151 | 0.41208 | 0.0807 | 0.496 | 0.878109 |
| cg02665568 | 0.0397 | 0.6025 | 0.9156 | -0.0746 | 0.5369 | 0.956114 | -0.0551 | 0.5872 | 0.878109 |
| cg03546163 | 0.0154 | 0.8468 | 0.9156 | -0.0303 | 0.8366 | 0.956114 | 0.0743 | 0.4578 | 0.878109 |
| cg06087101 | -0.1129 | 0.09849 | 0.7904 | -0.1248 | 0.3068 | 0.867733 | 0.0451 | 0.6037 | 0.878109 |
| cg07061368 | -0.0127 | 0.8722 | 0.9156 | -0.0317 | 0.8151 | 0.956114 | -0.0798 | 0.4227 | 0.878109 |
| cg07633853 | -0.037 | 0.6138 | 0.9156 | -0.2626 | 0.0283 | 0.41208 | -0.172 | 0.07264 | 0.878109 |
| cg08586216 | -0.0167 | 0.8784 | 0.9156 | 0.1918 | 0.3254 | 0.867733 | 0.0043 | 0.975 | 0.975 |
| cg10300814 | 0.0487 | 0.6327 | 0.9156 | -0.0059 | 0.9747 | 0.9852 | 0.0242 | 0.8557 | 0.94528 |
| cg10913456 | -0.0824 | 0.2729 | 0.9156 | -0.1859 | 0.1979 | 0.867733 | -0.0489 | 0.5884 | 0.878109 |
| cg14284211 | -0.0296 | 0.5765 | 0.9156 | 0.0247 | 0.7918 | 0.956114 | 0.0098 | 0.8862 | 0.94528 |
| cg14642437 | 0.0135 | 0.8462 | 0.9156 | -0.0527 | 0.6653 | 0.956114 | 0.1002 | 0.2648 | 0.878109 |
| cg16052510 | 0.0568 | 0.3671 | 0.9156 | -0.0764 | 0.4941 | 0.956114 | 0.0474 | 0.5502 | 0.878109 |
| cg17085721 | 0.0286 | 0.736 | 0.9156 | 0.0026 | 0.9852 | 0.9852 | 0.0953 | 0.3893 | 0.878109 |
| cg18726036 | 0.112 | 0.09913 | 0.7904 | -0.0578 | 0.6615 | 0.956114 | -0.0273 | 0.7471 | 0.919508 |
| cg19014730 | 0.1435 | 0.1482 | 0.7904 | -0.2024 | 0.2339 | 0.867733 | 0.1177 | 0.3461 | 0.878109 |

| GAD1 | | | | | | | | | |
| --- | --- | --- | --- | --- | --- | --- | --- | --- | --- |
|  | **Whole** | | | **Males** | | | **Females** | | |
| Probe | **Beta** | **P-value** | **FDR (Gene)** | **Beta** | **P-value** | **FDR (Gene)** | **Beta** | **P-value** | **FDR (Gene)** |
| cg00224929 | -0.1151 | 0.2565 | 0.582955 | -0.0017 | 0.9926 | 0.9926 | 0.089 | 0.4797 | 0.9953 |
| cg00729049 | -0.0639 | 0.3315 | 0.6375 | -0.0601 | 0.5836 | 0.892059 | -0.0209 | 0.7982 | 0.9953 |
| cg01089249 | 0.0021 | 0.9756 | 0.9756 | -0.3051 | 0.006905 | 0.172625 | 0.0619 | 0.4815 | 0.9953 |
| cg01089319 | -0.0659 | 0.1748 | 0.517222 | -0.1306 | 0.1276 | 0.556591 | -0.0415 | 0.4882 | 0.9953 |
| cg01763173 | 0.1152 | 0.144 | 0.517222 | -0.1679 | 0.2026 | 0.556591 | 0.0336 | 0.7518 | 0.9953 |
| cg02723395 | 0.0045 | 0.9298 | 0.968542 | -0.1428 | 0.111 | 0.556591 | 0.0019 | 0.9766 | 0.9953 |
| cg04105250 | 0.0493 | 0.4382 | 0.695313 | -0.0047 | 0.9669 | 0.9926 | 0.1051 | 0.1991 | 0.9953 |
| cg07420274 | -0.0169 | 0.7377 | 0.922125 | -0.0281 | 0.7614 | 0.964643 | 5.00E-04 | 0.9942 | 0.9953 |
| cg08863440 | -0.1331 | 0.006858 | 0.17145 | 0.0072 | 0.9323 | 0.9926 | -0.006 | 0.9258 | 0.9953 |
| cg09144707 | 0.0111 | 0.8725 | 0.968542 | 0.0548 | 0.6711 | 0.932083 | 0.0955 | 0.2618 | 0.9953 |
| cg11281641 | 0.0324 | 0.6359 | 0.922125 | -0.0311 | 0.8103 | 0.964643 | -0.0278 | 0.7345 | 0.9953 |
| cg11348701 | -0.1768 | 0.0494 | 0.30875 | -0.0967 | 0.5006 | 0.892059 | -0.0689 | 0.5692 | 0.9953 |
| cg11582100 | 0.0139 | 0.8867 | 0.968542 | -0.0867 | 0.6066 | 0.892059 | 0.0358 | 0.7731 | 0.9953 |
| cg14005211 | -0.0698 | 0.04549 | 0.30875 | -0.12 | 0.0499 | 0.425417 | -0.0434 | 0.3229 | 0.9953 |
| cg14486905 | -0.0578 | 0.1862 | 0.517222 | -0.0986 | 0.1816 | 0.556591 | 0.064 | 0.252 | 0.9953 |
| cg14914809 | -0.0296 | 0.7286 | 0.922125 | -0.184 | 0.2449 | 0.556591 | -0.0326 | 0.752 | 0.9953 |
| cg15126544 | 0.0712 | 0.1489 | 0.517222 | -0.1252 | 0.1661 | 0.556591 | 0.1095 | 0.06797 | 0.849625 |
| cg15306595 | -0.2275 | 0.03319 | 0.30875 | -0.117 | 0.5319 | 0.892059 | 0.0297 | 0.8302 | 0.9953 |
| cg15753746 | -0.1095 | 0.1203 | 0.517222 | 0.1533 | 0.167 | 0.556591 | 6.00E-04 | 0.9953 | 0.9953 |
| cg16911423 | 0.089 | 0.445 | 0.695313 | -0.0499 | 0.8094 | 0.964643 | 0.0822 | 0.5667 | 0.9953 |
| cg19009018 | 0.0927 | 0.2564 | 0.582955 | -0.1659 | 0.2248 | 0.556591 | 0.0544 | 0.6077 | 0.9953 |
| cg19538089 | -0.0079 | 0.9169 | 0.968542 | -0.0214 | 0.8637 | 0.981477 | 0.0853 | 0.3836 | 0.9953 |
| cg19846314 | -0.0378 | 0.3886 | 0.693929 | 0.1399 | 0.05105 | 0.425417 | -0.0109 | 0.8495 | 0.9953 |
| cg21535772 | -0.0975 | 0.3259 | 0.6375 | -0.0975 | 0.5732 | 0.892059 | 0.0456 | 0.7215 | 0.9953 |
| cg26391350 | -0.0384 | 0.6655 | 0.922125 | -0.0802 | 0.5764 | 0.892059 | 0.23 | 0.05303 | 0.849625 |

| HTR1A | | | | | | | | | |
| --- | --- | --- | --- | --- | --- | --- | --- | --- | --- |
|  | **Whole** | | | **Males** | | | **Females** | | |
| Probe | **Beta** | **P-value** | **FDR (Gene)** | **Beta** | **P-value** | **FDR (Gene)** | **Beta** | **P-value** | **FDR (Gene)** |
| cg01020744 | 0.0106 | 0.8343 | 0.903825 | 0.0908 | 0.3018 | 0.594425 | 0.0226 | 0.7247 | 0.817267 |
| cg02266732 | -0.0476 | 0.3816 | 0.674818 | 0.0273 | 0.792 | 0.812 | 0.0213 | 0.7544 | 0.817267 |
| cg04694812 | -0.1448 | 0.01779 | 0.23127 | -0.1747 | 0.07498 | 0.324913 | 0.0712 | 0.4038 | 0.61061 |
| cg04799838 | 0.1181 | 0.1346 | 0.660833 | 0.0964 | 0.4784 | 0.616318 | 0.138 | 0.1705 | 0.484714 |
| cg07839533 | -0.0996 | 0.1525 | 0.660833 | -0.1644 | 0.1596 | 0.5187 | -0.1075 | 0.2272 | 0.484714 |
| cg08259925 | 0.0332 | 0.571 | 0.674818 | 0.068 | 0.5215 | 0.616318 | 0.0904 | 0.2423 | 0.484714 |
| cg09698471 | 0.0487 | 0.477 | 0.674818 | -0.2473 | 0.03323 | 0.324913 | -0.0121 | 0.8856 | 0.8856 |
| cg10588470 | 0.0412 | 0.3416 | 0.674818 | 0.1407 | 0.06787 | 0.324913 | 0.0609 | 0.261 | 0.484714 |
| cg13666507 | -0.052 | 0.3586 | 0.674818 | -0.0714 | 0.4872 | 0.616318 | 0.1212 | 0.09676 | 0.484714 |
| cg15092168 | -0.0382 | 0.521 | 0.674818 | 0.0844 | 0.3658 | 0.594425 | -0.0968 | 0.2391 | 0.484714 |
| cg16280141 | -0.0454 | 0.4468 | 0.674818 | -0.1288 | 0.2331 | 0.594425 | 0.2164 | 0.003174 | **0.041262** |
| cg23448729 | 0.0521 | 0.3248 | 0.674818 | 0.0914 | 0.3227 | 0.594425 | -0.0507 | 0.4421 | 0.61061 |
| cg27615388 | 0.0017 | 0.9608 | 0.9608 | -0.0139 | 0.812 | 0.812 | 0.0314 | 0.4697 | 0.61061 |

| HTR2A | | | | | | | | | |
| --- | --- | --- | --- | --- | --- | --- | --- | --- | --- |
|  | **Whole** | | | **Males** | | | **Females** | | |
| Probe | **Beta** | **P-value** | **FDR (Gene)** | **Beta** | **P-value** | **FDR (Gene)** | **Beta** | **P-value** | **FDR (Gene)** |
| cg00308665 | 0.0488 | 0.4868 | 0.9857 | -0.0733 | 0.5726 | 0.68712 | -0.0276 | 0.7543 | 0.9396 |
| cg02250787 | -0.0416 | 0.5508 | 0.9857 | -0.2273 | 0.07555 | 0.210432 | 0.0146 | 0.8669 | 0.9396 |
| cg06476131 | -0.013 | 0.8378 | 0.9857 | -0.316 | 0.004871 | **0.029226** | 0.1765 | 0.02728 | 0.1761 |
| cg09361691 | -0.1354 | 0.03219 | 0.38628 | -0.3527 | 0.001296 | **0.015552** | 0.0408 | 0.6169 | 0.9396 |
| cg11514288 | 0.0015 | 0.9795 | 0.9857 | -0.1098 | 0.2933 | 0.43995 | 0.0407 | 0.5913 | 0.9396 |
| cg12089079 | -0.0781 | 0.2738 | 0.8214 | -0.2426 | 0.08768 | 0.210432 | -0.0283 | 0.7408 | 0.9396 |
| cg12367389 | 0.0055 | 0.9411 | 0.9857 | -0.0061 | 0.9661 | 0.9661 | -0.0068 | 0.9396 | 0.9396 |
| cg14059288 | -0.1516 | 0.1409 | 0.7188 | -0.2898 | 0.1119 | 0.2238 | 0.2735 | 0.02935 | 0.1761 |
| cg15894389 | -0.0171 | 0.7837 | 0.9857 | -0.1105 | 0.2905 | 0.43995 | 0.0515 | 0.5294 | 0.9396 |
| cg16188532 | -0.0779 | 0.1797 | 0.7188 | -0.2064 | 0.03577 | 0.14308 | -0.0227 | 0.7607 | 0.9396 |
| cg20102280 | -0.0093 | 0.8987 | 0.9857 | -0.0619 | 0.6432 | 0.701673 | 0.1157 | 0.2079 | 0.8316 |
| cg26950475 | -0.0013 | 0.9857 | 0.9857 | -0.0998 | 0.3847 | 0.512933 | -0.0196 | 0.8505 | 0.9396 |

| NPSR1 | | | | | | | | | |
| --- | --- | --- | --- | --- | --- | --- | --- | --- | --- |
|  | **Whole** | | | **Males** | | | **Females** | | |
| Probe | **Beta** | **P-value** | **FDR (Gene)** | **Beta** | **P-value** | **FDR (Gene)** | **Beta** | **P-value** | **FDR (Gene)** |
| cg00081087 | -0.0708 | 0.3385 | 0.866125 | -0.0697 | 0.5941 | 0.965413 | -0.0105 | 0.9111 | 0.9111 |
| cg03382549 | 0.1207 | 0.1725 | 0.866125 | 0.0056 | 0.9706 | 0.9706 | 0.2105 | 0.05748 | 0.24908 |
| cg05399607 | 0.0043 | 0.9622 | 0.9622 | -0.21 | 0.1605 | 0.6435 | 0.1293 | 0.2702 | 0.71916 |
| cg06506864 | 0.0469 | 0.3728 | 0.866125 | -0.0798 | 0.3933 | 0.748614 | 0.1704 | 0.007977 | 0.09581 |
| cg08251685 | -0.0691 | 0.4108 | 0.866125 | -0.0245 | 0.8733 | 0.9706 | 0.0175 | 0.8675 | 0.9111 |
| cg15754660 | 0.0855 | 0.09754 | 0.866125 | 0.1037 | 0.2475 | 0.6435 | 0.071 | 0.2766 | 0.71916 |
| cg17744825 | -0.0339 | 0.722 | 0.9386 | 0.0176 | 0.9133 | 0.9706 | 0.119 | 0.3397 | 0.736017 |
| cg19194095 | 0.0039 | 0.9622 | 0.9622 | -0.1729 | 0.2049 | 0.6435 | 0.0873 | 0.4303 | 0.799129 |
| cg20495677 | 0.0635 | 0.3644 | 0.866125 | 0.0068 | 0.9536 | 0.9706 | 0.0213 | 0.8139 | 0.9111 |
| cg20842782 | -0.0159 | 0.8542 | 0.9622 | 0.1281 | 0.4031 | 0.748614 | 0.0262 | 0.8106 | 0.9111 |
| cg23448390 | -0.0662 | 0.533 | 0.866125 | -0.4318 | 0.02289 | 0.29757 | 0.0512 | 0.6924 | 0.9111 |
| cg23862011 | 0.0454 | 0.6039 | 0.8723 | -0.0116 | 0.9414 | 0.9706 | 0.038 | 0.7336 | 0.9111 |
| cg24929847 | 0.0733 | 0.4825 | 0.866125 | -0.3039 | 0.08601 | 0.559065 | 0.3266 | 0.01474 | 0.09581 |

| OXTR | | | | | | | | | |
| --- | --- | --- | --- | --- | --- | --- | --- | --- | --- |
|  | **Whole** | | | **Males** | | | **Females** | | |
| Probe | **Beta** | **P-value** | **FDR (Gene)** | **Beta** | **P-value** | **FDR (Gene)** | **Beta** | **P-value** | **FDR (Gene)** |
| cg00078085 | -0.0551 | 0.3384 | 0.661556 | 0.0346 | 0.7427 | 0.881282 | -0.0451 | 0.5208 | 0.9555 |
| cg00385883 | 0.162 | 0.1131 | 0.661556 | -0.0794 | 0.6432 | 0.881282 | 0.3078 | 0.02286 | 0.169975 |
| cg02192228 | 0.088 | 0.1461 | 0.661556 | 0.0399 | 0.6974 | 0.881282 | -0.0012 | 0.9884 | 0.9884 |
| cg03987506 | -0.0473 | 0.4157 | 0.661556 | 0.0186 | 0.8568 | 0.9273 | -0.0361 | 0.6191 | 0.9555 |
| cg04523291 | 0.0508 | 0.3698 | 0.661556 | -0.0093 | 0.9273 | 0.9273 | 0.1559 | 0.02615 | 0.169975 |
| cg08535600 | 0 | 0.9995 | 0.9995 | -0.1099 | 0.2265 | 0.881282 | 0.0181 | 0.7881 | 0.9555 |
| cg09353063 | 0.0666 | 0.2741 | 0.661556 | 0.0388 | 0.7329 | 0.881282 | 0.0472 | 0.541 | 0.9555 |
| cg12695586 | -0.0267 | 0.6667 | 0.733092 | -0.1694 | 0.1618 | 0.881282 | 0.0337 | 0.6621 | 0.9555 |
| cg15317815 | 0.0197 | 0.6767 | 0.733092 | 0.034 | 0.6542 | 0.881282 | 0.1222 | 0.05432 | 0.235387 |
| cg17285225 | -0.0627 | 0.2339 | 0.661556 | -0.0337 | 0.7149 | 0.881282 | -0.0261 | 0.6983 | 0.9555 |
| cg19619174 | 0.0986 | 0.2742 | 0.661556 | -0.2726 | 0.07895 | 0.881282 | 0.1683 | 0.132 | 0.429 |
| cg23391006 | -0.0601 | 0.458 | 0.661556 | 0.048 | 0.7457 | 0.881282 | -0.0249 | 0.8162 | 0.9555 |
| cg27501759 | -0.0199 | 0.5233 | 0.68029 | 0.0178 | 0.7278 | 0.881282 | 0.0061 | 0.882 | 0.9555 |

| SGK1 | | | | | | | | | |
| --- | --- | --- | --- | --- | --- | --- | --- | --- | --- |
|  | **Whole** | | | **Males** | | | **Females** | | |
| Probe | **Beta** | **P-value** | **FDR (Gene)** | **Beta** | **P-value** | **FDR (Gene)** | **Beta** | **P-value** | **FDR (Gene)** |
| cg00959636 | -0.3081 | 0.000787 | **0.035424** | -0.0941 | 0.5429 | 0.939635 | -0.0021 | 0.9856 | 0.9856 |
| cg01059669 | -0.1247 | 0.1967 | 0.5901 | 0.1557 | 0.337 | 0.871816 | -0.1666 | 0.1743 | 0.6678 |
| cg02904344 | 0.0517 | 0.5148 | 0.858 | 0.1559 | 0.2766 | 0.871816 | 0.0453 | 0.6538 | 0.8406 |
| cg03146155 | 0.0765 | 0.2543 | 0.6585 | -0.0719 | 0.5347 | 0.939635 | -0.0878 | 0.2901 | 0.713045 |
| cg03400131 | -0.0144 | 0.8348 | 0.9684 | 0.02 | 0.8799 | 0.960239 | 0.0422 | 0.6169 | 0.824824 |
| cg03762694 | -0.1551 | 0.05531 | 0.3957 | 0.1272 | 0.3681 | 0.871816 | -0.1048 | 0.3082 | 0.713045 |
| cg03944089 | 0.0093 | 0.8968 | 0.9684 | -0.0517 | 0.6711 | 0.953743 | 0.0929 | 0.3133 | 0.713045 |
| cg04060943 | 0.0431 | 0.6092 | 0.877091 | -0.1797 | 0.2277 | 0.871816 | 0.0985 | 0.3486 | 0.713045 |
| cg04905719 | -0.0536 | 0.3023 | 0.680175 | -0.0263 | 0.7418 | 0.953743 | -0.085 | 0.2081 | 0.6678 |
| cg05183646 | -0.0835 | 0.1623 | 0.521679 | -0.2501 | 0.02184 | 0.480488 | -0.0899 | 0.2205 | 0.6678 |
| cg05966641 | 0.0029 | 0.9684 | 0.9684 | -0.2313 | 0.07036 | 0.63324 | -0.0256 | 0.7723 | 0.923566 |
| cg06358608 | -0.0329 | 0.6432 | 0.877091 | 0.0762 | 0.5397 | 0.939635 | 0.0724 | 0.427 | 0.7686 |
| cg06642177 | 0.0945 | 0.007257 | 0.163283 | 0.0159 | 0.7968 | 0.960239 | 0.0319 | 0.494 | 0.793929 |
| cg06849960 | -0.1124 | 0.2532 | 0.6585 | -0.2079 | 0.2853 | 0.871816 | -0.1347 | 0.2537 | 0.713045 |
| cg07340870 | 0.2117 | 0.05854 | 0.3957 | 0.2113 | 0.2364 | 0.871816 | 0.2343 | 0.1282 | 0.6678 |
| cg08239804 | 0.0299 | 0.7262 | 0.961147 | -0.297 | 0.03905 | 0.480488 | -0.0556 | 0.6232 | 0.824824 |
| cg08550353 | 0.0321 | 0.6409 | 0.877091 | 0.1066 | 0.3565 | 0.871816 | 0.0638 | 0.468 | 0.788167 |
| cg08640361 | 0.1429 | 0.1342 | 0.50325 | 0.0185 | 0.9075 | 0.960239 | -0.1126 | 0.3455 | 0.713045 |
| cg08647910 | -0.0548 | 0.4741 | 0.826442 | -5.00E-04 | 0.9974 | 0.9974 | -0.0064 | 0.9465 | 0.9856 |
| cg08698685 | 0.0084 | 0.8757 | 0.9684 | -0.0367 | 0.6733 | 0.953743 | 0.0174 | 0.8055 | 0.929423 |
| cg09315391 | -0.004 | 0.9668 | 0.9684 | -0.112 | 0.447 | 0.939635 | 0.0928 | 0.4729 | 0.788167 |
| cg09404376 | 0.1271 | 0.0718 | 0.3957 | 0.101 | 0.416 | 0.936 | 0.1743 | 0.05934 | 0.5841 |
| cg09872934 | -0.0159 | 0.8251 | 0.9684 | -0.1379 | 0.2846 | 0.871816 | -0.0184 | 0.8376 | 0.9423 |
| cg10105971 | -0.0449 | 0.6336 | 0.877091 | -0.0271 | 0.8566 | 0.960239 | -0.0832 | 0.5142 | 0.797897 |
| cg11856561 | 0.0101 | 0.9266 | 0.9684 | -0.2646 | 0.1481 | 0.833063 | 0.1756 | 0.2106 | 0.6678 |
| cg12009778 | 0.0321 | 0.7607 | 0.9684 | -0.3621 | 0.04271 | 0.480488 | 0.2384 | 0.07788 | 0.5841 |
| cg13307058 | 0.1 | 0.09686 | 0.43587 | 0.0762 | 0.5068 | 0.939635 | 0.0021 | 0.9774 | 0.9856 |
| cg14905466 | 0.0363 | 0.5627 | 0.877091 | 0.0397 | 0.7217 | 0.953743 | 0.0038 | 0.9607 | 0.9856 |
| cg17284168 | -0.0591 | 0.5934 | 0.877091 | 0.0893 | 0.6447 | 0.953743 | -0.0827 | 0.5454 | 0.8181 |
| cg17689707 | -0.1106 | 0.158 | 0.521679 | 0.0208 | 0.8779 | 0.960239 | -0.0498 | 0.6197 | 0.824824 |
| cg18566177 | 0.0087 | 0.9256 | 0.9684 | -0.012 | 0.9389 | 0.960239 | 0.0361 | 0.7638 | 0.923566 |
| cg20393620 | -0.0121 | 0.8861 | 0.9684 | 0.0443 | 0.7363 | 0.953743 | 0.0178 | 0.8794 | 0.965195 |
| cg20655113 | 0.0267 | 0.7832 | 0.9684 | 0.0691 | 0.7113 | 0.953743 | 0.1472 | 0.2075 | 0.6678 |
| cg20822858 | -0.0811 | 0.4775 | 0.826442 | 0.0205 | 0.9191 | 0.960239 | 0.169 | 0.2226 | 0.6678 |
| cg21064939 | -0.1945 | 0.04844 | 0.3957 | -0.2798 | 0.1248 | 0.833063 | -0.3021 | 0.0128 | 0.192 |
| cg21078322 | 0.0748 | 0.3227 | 0.6915 | -0.0365 | 0.7874 | 0.960239 | -0.085 | 0.3702 | 0.724304 |
| cg21366688 | 0.0704 | 0.3438 | 0.703227 | -0.0606 | 0.627 | 0.953743 | 0.1606 | 0.09767 | 0.627879 |
| cg21676440 | -0.1223 | 0.2896 | 0.680175 | -0.2162 | 0.2948 | 0.871816 | -0.2585 | 0.07001 | 0.5841 |
| cg21834463 | 0.037 | 0.4706 | 0.826442 | -0.0832 | 0.3456 | 0.871816 | -0.1629 | 0.01225 | 0.192 |
| cg23347562 | 0.0789 | 0.457 | 0.826442 | -0.0389 | 0.8407 | 0.960239 | 0.1646 | 0.2177 | 0.6678 |
| cg24688636 | -0.1439 | 0.06378 | 0.3957 | -0.2034 | 0.1302 | 0.833063 | -0.2642 | 0.006513 | 0.192 |
| cg25025235 | -0.0972 | 0.2634 | 0.6585 | -0.095 | 0.5276 | 0.939635 | -0.0643 | 0.5671 | 0.82321 |
| cg25661219 | -0.156 | 0.07914 | 0.3957 | -0.3501 | 0.03569 | 0.480488 | -0.0299 | 0.7799 | 0.923566 |
| cg26557834 | 0.1684 | 0.03655 | 0.3957 | 0.1328 | 0.3486 | 0.871816 | 0.1004 | 0.3188 | 0.713045 |
| cg27289153 | 0.1291 | 0.1258 | 0.50325 | -0.0731 | 0.6134 | 0.953743 | 0.0838 | 0.423 | 0.7686 |

| TMEM132D | | | | | | | | | |
| --- | --- | --- | --- | --- | --- | --- | --- | --- | --- |
|  | **Whole** | | | **Males** | | | **Females** | | |
| Probe | **Beta** | **P-value** | **FDR (Gene)** | **Beta** | **P-value** | **FDR (Gene)** | **Beta** | **P-value** | **FDR (Gene)** |
| cg01163404 | 0.1089 | 0.1225 | 0.601033 | 0.2281 | 0.05266 | 0.410459 | 0.0125 | 0.8917 | 0.9854 |
| cg01202700 | 0.2627 | 0.01158 | 0.211335 | 0.3553 | 0.05984 | 0.410459 | 0.2938 | 0.02614 | 0.432744 |
| cg01831527 | -0.0222 | 0.7099 | 0.94071 | 0.0595 | 0.5857 | 0.92345 | -0.0815 | 0.2604 | 0.746164 |
| cg02365079 | 0.1364 | 0.04654 | 0.283118 | 0.2054 | 0.08221 | 0.500111 | 0.0107 | 0.9031 | 0.9854 |
| cg02767665 | 0.0241 | 0.7505 | 0.94071 | 0.0209 | 0.8775 | 0.957934 | 0.0881 | 0.3673 | 0.812512 |
| cg03283235 | -0.0326 | 0.6895 | 0.94071 | 0.0119 | 0.9325 | 0.9775 | -0.0019 | 0.9854 | 0.9854 |
| cg03420866 | -0.0508 | 0.537 | 0.94071 | 0.1719 | 0.2448 | 0.770758 | -0.0181 | 0.8623 | 0.9854 |
| cg03469054 | 0.0441 | 0.4531 | 0.94071 | -0.0715 | 0.5112 | 0.92345 | 0.1036 | 0.1493 | 0.746164 |
| cg03685843 | -0.0021 | 0.9685 | 0.981951 | -0.1253 | 0.2045 | 0.746425 | -0.0747 | 0.2763 | 0.746164 |
| cg04386563 | -0.0081 | 0.8993 | 0.976613 | -0.117 | 0.2773 | 0.778573 | 0.0609 | 0.4494 | 0.849815 |
| cg04414975 | -0.081 | 0.3413 | 0.94071 | -0.307 | 0.02555 | 0.410459 | -0.0691 | 0.5355 | 0.849815 |
| cg04729491 | 0.0398 | 0.7088 | 0.94071 | -0.0291 | 0.8782 | 0.957934 | -0.0106 | 0.9377 | 0.9854 |
| cg04925956 | 0.0389 | 0.5332 | 0.94071 | -0.0241 | 0.8394 | 0.957934 | -0.0518 | 0.4992 | 0.849815 |
| cg05160910 | 0.0375 | 0.6074 | 0.94071 | 0.2321 | 0.06185 | 0.410459 | 0.0973 | 0.3156 | 0.794441 |
| cg05384697 | -0.1853 | 0.04234 | 0.280984 | -0.1445 | 0.4073 | 0.838892 | -0.1897 | 0.08699 | 0.58473 |
| cg05479657 | 0.0645 | 0.4126 | 0.94071 | -0.0498 | 0.6908 | 0.92345 | -0.0398 | 0.6969 | 0.97721 |
| cg05742082 | -0.0227 | 0.7898 | 0.960923 | -0.0993 | 0.564 | 0.92345 | 0.0962 | 0.3383 | 0.796642 |
| cg06200996 | -0.0308 | 0.5584 | 0.94071 | -0.0505 | 0.5793 | 0.92345 | -0.0432 | 0.5236 | 0.849815 |
| cg06679878 | -0.0165 | 0.8482 | 0.967478 | -0.3303 | 0.02196 | 0.410459 | -0.2043 | 0.06154 | 0.546364 |
| cg07056260 | -0.0643 | 0.3118 | 0.94071 | -0.1391 | 0.1953 | 0.746425 | -0.0224 | 0.7859 | 0.97721 |
| cg07067993 | -0.0376 | 0.4332 | 0.94071 | -0.0713 | 0.3777 | 0.838892 | 0.0873 | 0.1595 | 0.746164 |
| cg07230440 | 0.0776 | 0.1235 | 0.601033 | 0.0329 | 0.7351 | 0.941444 | -0.0527 | 0.3882 | 0.819269 |
| cg07350016 | 0.0513 | 0.5975 | 0.94071 | -0.0794 | 0.6195 | 0.92345 | 0.039 | 0.7624 | 0.97721 |
| cg08261450 | 0.0405 | 0.5438 | 0.94071 | -0.1287 | 0.2721 | 0.778573 | -0.0408 | 0.6287 | 0.936635 |
| cg08546107 | -0.0013 | 0.9872 | 0.9872 | -0.1925 | 0.2152 | 0.748076 | 0.1208 | 0.2362 | 0.746164 |
| cg09044656 | 0.0068 | 0.9204 | 0.976613 | 0.0904 | 0.4137 | 0.838892 | 0.0965 | 0.2862 | 0.746164 |
| cg10639585 | 0.028 | 0.6388 | 0.94071 | 0.046 | 0.6713 | 0.92345 | 0.0061 | 0.9347 | 0.9854 |
| cg11023224 | 0.0052 | 0.9411 | 0.981433 | -0.1195 | 0.3893 | 0.838892 | 0.1089 | 0.1996 | 0.746164 |
| cg11160362 | 0.108 | 0.6069 | 0.94071 | 0.0122 | 0.9699 | 0.9775 | -0.2488 | 0.3928 | 0.819269 |
| cg11230248 | 0.0825 | 0.2952 | 0.94071 | 0.1943 | 0.1686 | 0.733865 | 0.0137 | 0.89 | 0.9854 |
| cg11496226 | -0.1054 | 0.02166 | 0.225883 | -0.0574 | 0.4759 | 0.890787 | -0.1544 | 0.006169 | 0.225169 |
| cg12072740 | -0.0412 | 0.5541 | 0.94071 | -0.2433 | 0.04073 | 0.410459 | -0.0985 | 0.266 | 0.746164 |
| cg12820134 | 0.0178 | 0.8193 | 0.967478 | -0.1296 | 0.3286 | 0.814923 | -0.0641 | 0.529 | 0.849815 |
| cg13090220 | 0.1599 | 0.04151 | 0.280984 | 0.1352 | 0.3276 | 0.814923 | 0.1136 | 0.2506 | 0.746164 |
| cg13123585 | -0.0419 | 0.5674 | 0.94071 | -0.1381 | 0.2477 | 0.770758 | 0.003 | 0.9756 | 0.9854 |
| cg13916352 | -0.0723 | 0.4593 | 0.94071 | -0.1895 | 0.2534 | 0.770758 | -0.0561 | 0.6555 | 0.93898 |
| cg14504768 | -0.0147 | 0.8417 | 0.967478 | 0.071 | 0.5863 | 0.92345 | 0.0547 | 0.5513 | 0.856274 |
| cg14918019 | 0.0365 | 0.6602 | 0.94071 | -0.0041 | 0.9775 | 0.9775 | 0.0093 | 0.9281 | 0.9854 |
| cg15617706 | 0.2868 | 0.005854 | 0.142447 | 0.0345 | 0.8459 | 0.957934 | 0.2738 | 0.0431 | 0.524383 |
| cg15936861 | 0.0317 | 0.718 | 0.94071 | -0.2418 | 0.1079 | 0.562621 | 0.1396 | 0.2007 | 0.746164 |
| cg16048915 | 0.056 | 0.4032 | 0.94071 | -0.0314 | 0.788 | 0.957934 | 0.0605 | 0.4764 | 0.849815 |
| cg16533379 | 0.0551 | 0.3138 | 0.94071 | 0.0192 | 0.8424 | 0.957934 | 0.0035 | 0.9588 | 0.9854 |
| cg17157798 | -0.0147 | 0.8296 | 0.967478 | -0.1897 | 0.1064 | 0.562621 | 0.0837 | 0.3527 | 0.804597 |
| cg17186073 | -0.0772 | 0.09916 | 0.556822 | -0.112 | 0.1709 | 0.733865 | -0.047 | 0.4626 | 0.849815 |
| cg17444697 | 0.0559 | 0.3957 | 0.94071 | 0.0451 | 0.7068 | 0.92345 | 0.0866 | 0.2815 | 0.746164 |
| cg17513770 | 0.0176 | 0.7401 | 0.94071 | -0.0047 | 0.9605 | 0.9775 | 0.104 | 0.1184 | 0.720267 |
| cg17718276 | -0.0548 | 0.3384 | 0.94071 | 0.2271 | 0.04171 | 0.410459 | -0.0024 | 0.972 | 0.9854 |
| cg17735631 | 0.0077 | 0.9231 | 0.976613 | 0.1239 | 0.3474 | 0.818071 | 0.0746 | 0.4832 | 0.849815 |
| cg17883960 | 0.031 | 0.6779 | 0.94071 | -0.0057 | 0.9644 | 0.9775 | 0.0627 | 0.5133 | 0.849815 |
| cg18180056 | 0.0718 | 0.4296 | 0.94071 | -0.1 | 0.5439 | 0.92345 | 0.0359 | 0.7525 | 0.97721 |
| cg18437033 | -0.0617 | 0.5145 | 0.94071 | -0.0232 | 0.8792 | 0.957934 | 0.0698 | 0.5676 | 0.863225 |
| cg18723572 | 0.166 | 0.01526 | 0.214377 | 0.0948 | 0.4355 | 0.853908 | 0.2139 | 0.01315 | 0.319983 |
| cg18758559 | 0.0026 | 0.9648 | 0.981951 | 0.0885 | 0.4003 | 0.838892 | -0.0786 | 0.2807 | 0.746164 |
| cg19070138 | 0.1849 | 0.001056 | 0.077088 | 0.1522 | 0.1168 | 0.568427 | -0.0032 | 0.9649 | 0.9854 |
| cg19700087 | 0.0244 | 0.6669 | 0.94071 | 0.0375 | 0.7084 | 0.92345 | 0.1107 | 0.1331 | 0.746164 |
| cg19790509 | 0.0827 | 0.2906 | 0.94071 | -0.0765 | 0.5785 | 0.92345 | 0.0718 | 0.4571 | 0.849815 |
| cg20168964 | 0.0867 | 0.1801 | 0.730406 | 0.027 | 0.8083 | 0.957934 | -0.0264 | 0.7513 | 0.97721 |
| cg20327057 | 0.2712 | 0.003043 | 0.11107 | 0.0846 | 0.6043 | 0.92345 | 0.2435 | 0.02964 | 0.432744 |
| cg20470734 | 0.048 | 0.4505 | 0.94071 | -0.194 | 0.05933 | 0.410459 | 0.0168 | 0.8443 | 0.9854 |
| cg21903395 | -0.0221 | 0.6757 | 0.94071 | 0.0363 | 0.7003 | 0.92345 | 0.0197 | 0.7755 | 0.97721 |
| cg23266743 | -0.0606 | 0.4741 | 0.94071 | -0.0486 | 0.7506 | 0.944721 | -0.0156 | 0.8861 | 0.9854 |
| cg23733052 | 0.19 | 0.03625 | 0.280984 | -0.1412 | 0.3326 | 0.814923 | 0.0758 | 0.5326 | 0.849815 |
| cg23805623 | 0.0288 | 0.5975 | 0.94071 | -0.181 | 0.06041 | 0.410459 | 0.0655 | 0.3302 | 0.796642 |
| cg23917477 | -0.0209 | 0.7603 | 0.94071 | 0.0514 | 0.6656 | 0.92345 | 0.2477 | 0.004213 | 0.225169 |
| cg24008358 | 0.0659 | 0.1666 | 0.7154 | 0.1575 | 0.06094 | 0.410459 | -0.0162 | 0.7898 | 0.97721 |
| cg25015139 | -0.0301 | 0.6985 | 0.94071 | -0.1862 | 0.1872 | 0.746425 | 0.1165 | 0.231 | 0.746164 |
| cg25102216 | 0.1816 | 0.02538 | 0.231593 | -0.029 | 0.8573 | 0.957934 | 0.1068 | 0.2708 | 0.746164 |
| cg25625370 | 0.0714 | 0.1421 | 0.648331 | 0.0379 | 0.6591 | 0.92345 | 0.1122 | 0.06271 | 0.546364 |
| cg26322591 | -0.0173 | 0.7326 | 0.94071 | -0.2447 | 0.009236 | 0.410459 | 0.1045 | 0.08811 | 0.58473 |
| cg26364947 | 0.0555 | 0.2 | 0.768421 | -0.0056 | 0.9423 | 0.9775 | 0.0241 | 0.656 | 0.93898 |
| cg26411747 | 0.2294 | 0.01762 | 0.214377 | 0.1354 | 0.4445 | 0.853908 | 0.1603 | 0.1791 | 0.746164 |
| cg26614129 | 0.0106 | 0.8801 | 0.976613 | 0.0496 | 0.686 | 0.92345 | -0.1679 | 0.06736 | 0.546364 |
| cg27463181 | 0.0093 | 0.892 | 0.976613 | 0.1093 | 0.3349 | 0.814923 | -0.031 | 0.7374 | 0.97721 |

**Figure S1:**

Multidimensional scaling (MDS) plots used to investigate population structure in the discovery sample (a) and replication sample (b).

**a)**

**b)**

**Figure S2: QQ plots of p-values for the discovery sample**

Theoretical vs observed distributions for all the 424,834 p-values from the case-control analysis, for the whole sample (a), males only (b) and females only (c).

**a)**

**b)**

**c)**

**Figure S3: QQ plots of p-values for the replication sample**

Theoretical vs observed distributions for all 425,119 p-values from the case-control analysis, for the whole sample (a), males only (b) and females only (c).

**a)**

**b)**

**c)**

**Figure S4:**

Box plots of DNA methylation levels for the significant CpGs in the gene-targeted analysis in discovery and replication sample in females.

**
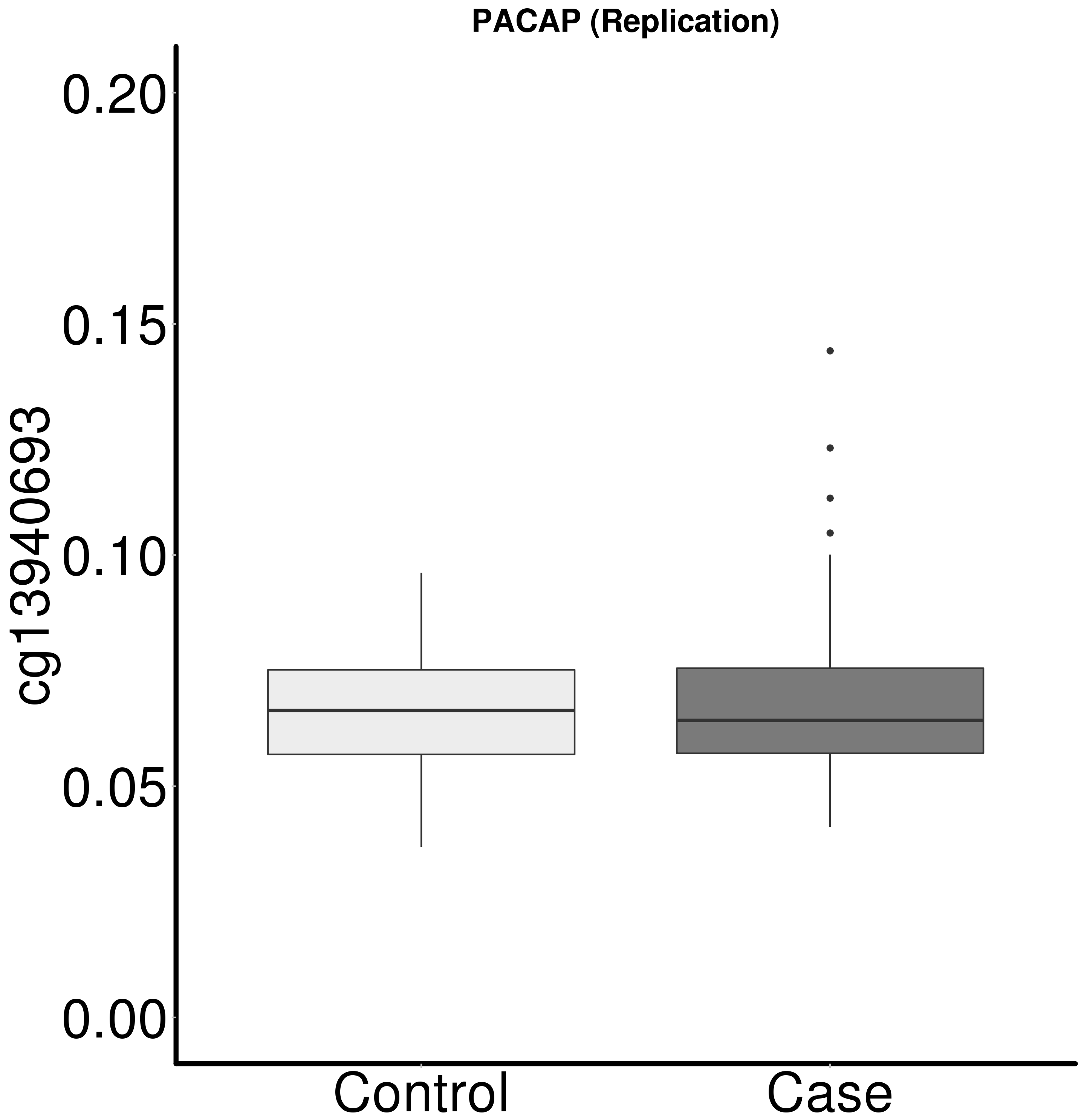

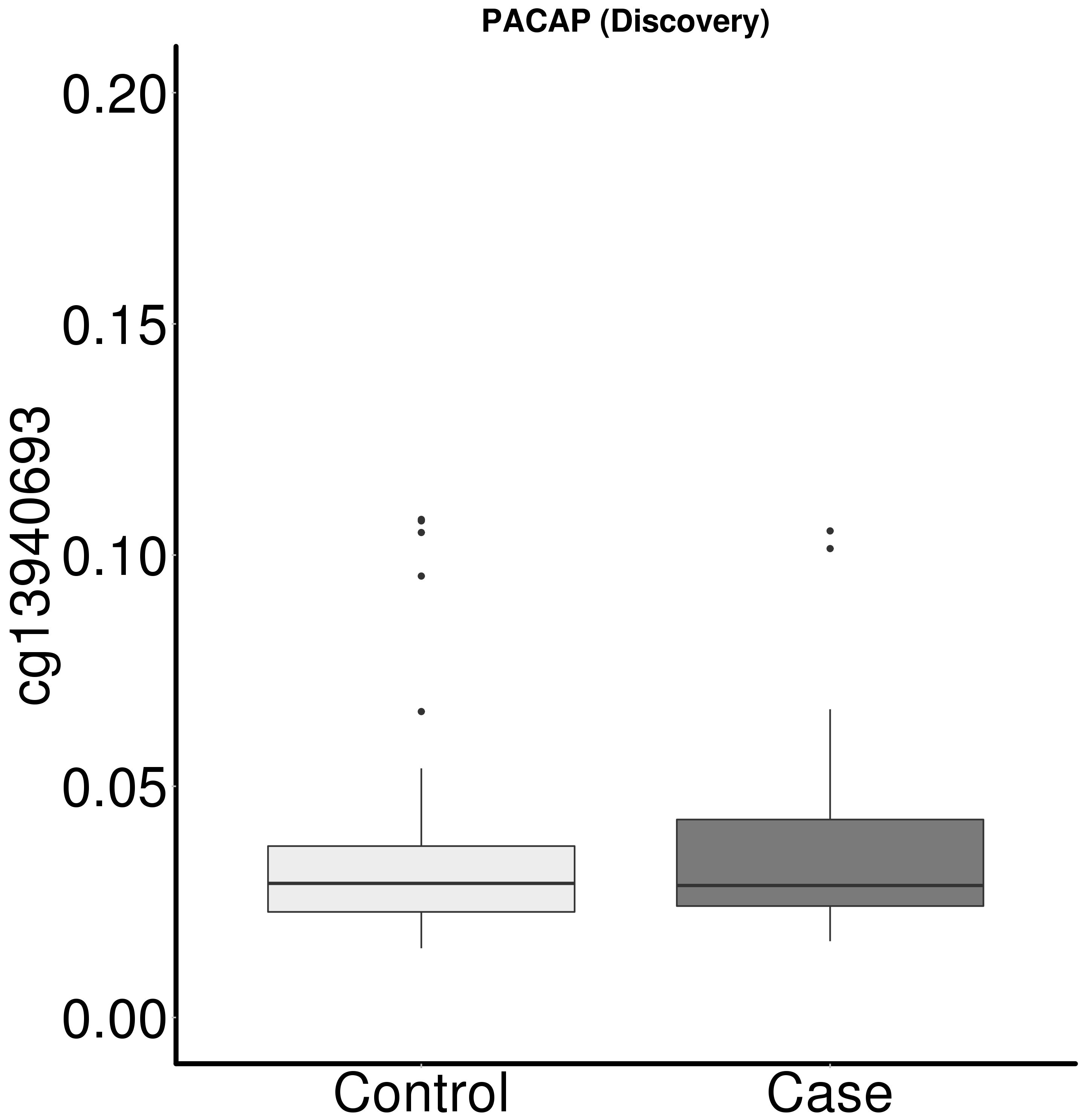
**

**Figure S5:**

Box plots of DNA methylation levels for the significant CpGs in the gene-targeted analysis in discovery and replication sample in the whole sample.

**
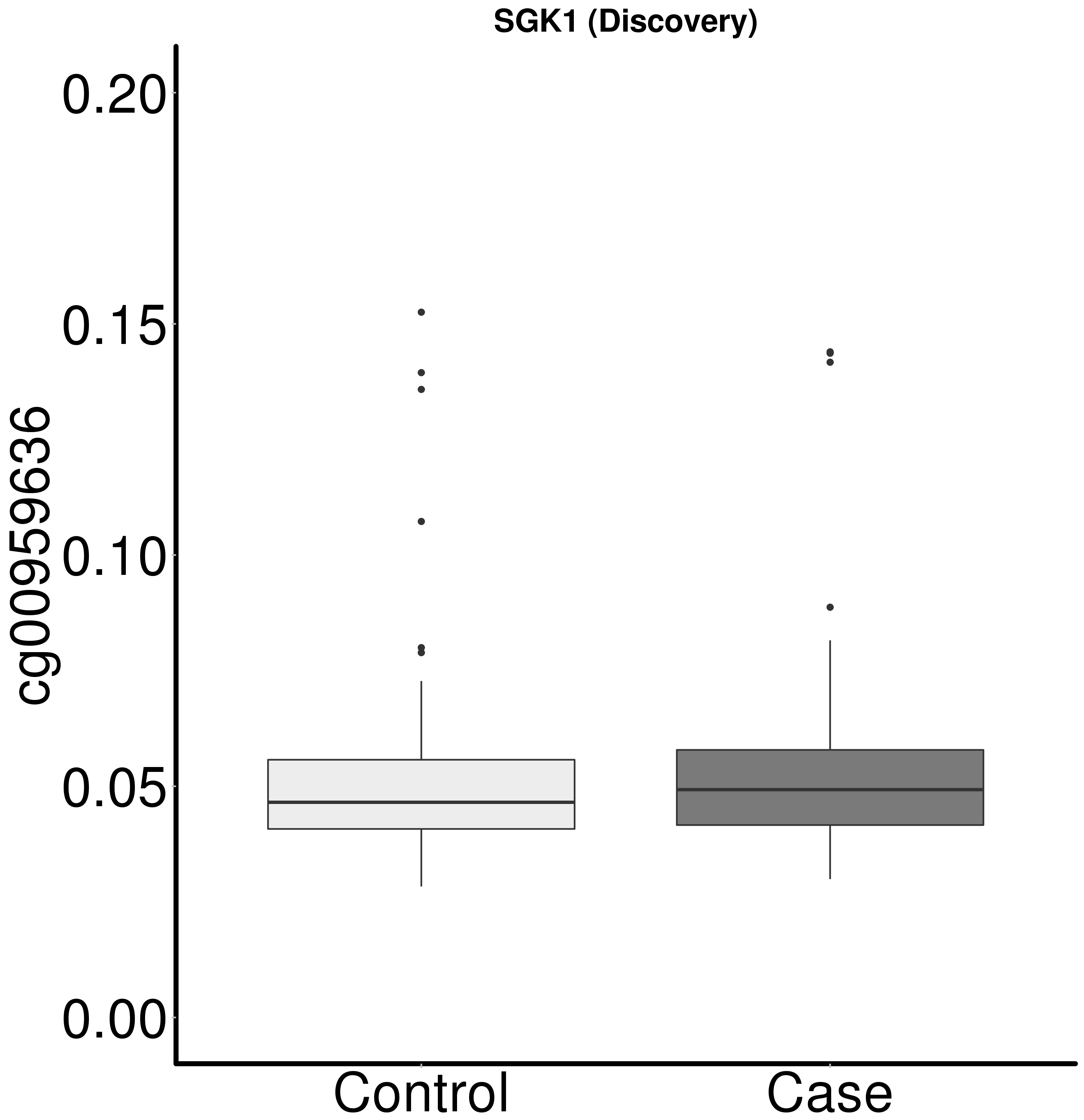

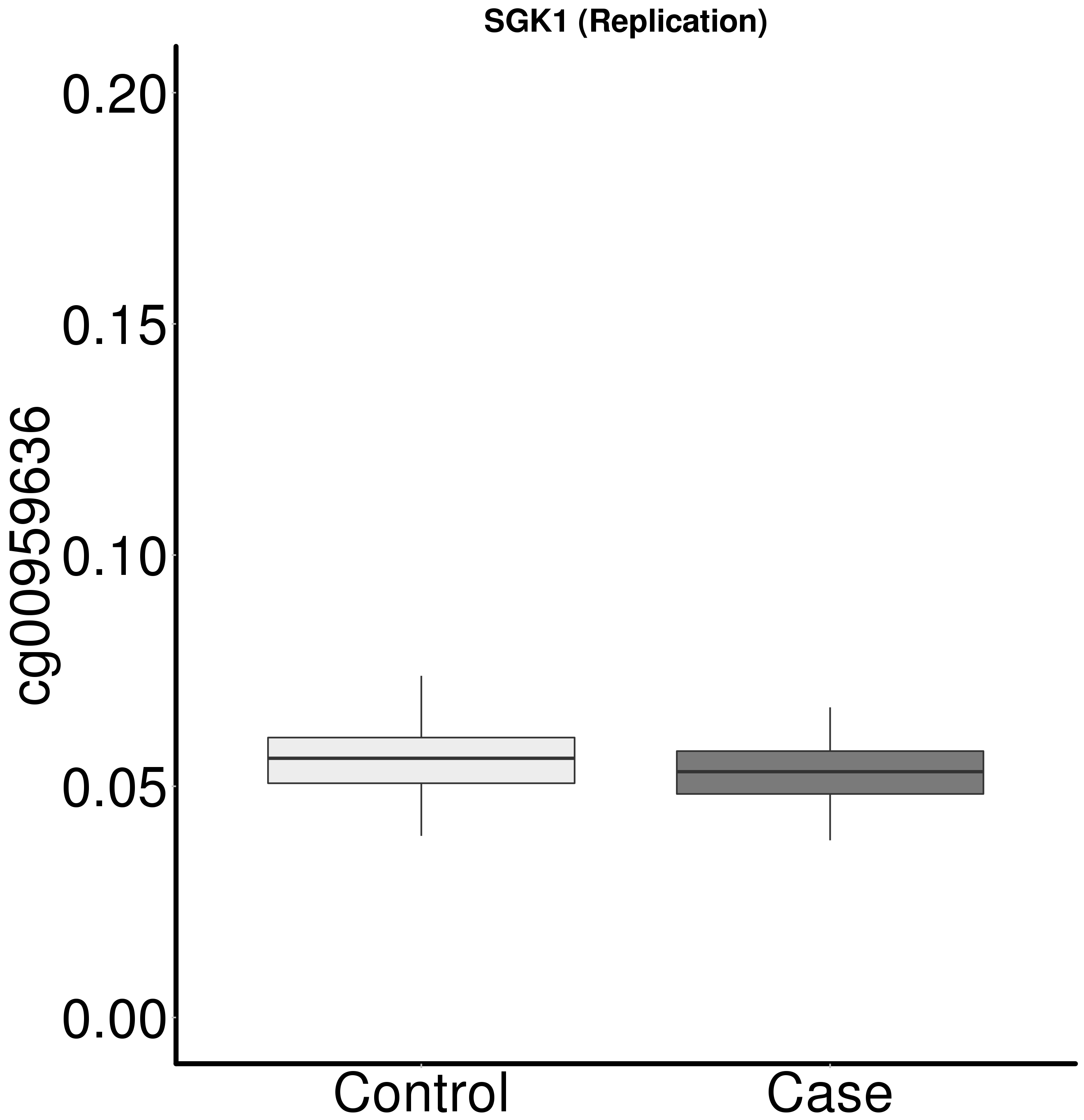
**

**Figure S6:**

Box plots of DNA methylation levels for the significant CpGs in the gene-targeted analysis in discovery and replication sample in males.

**
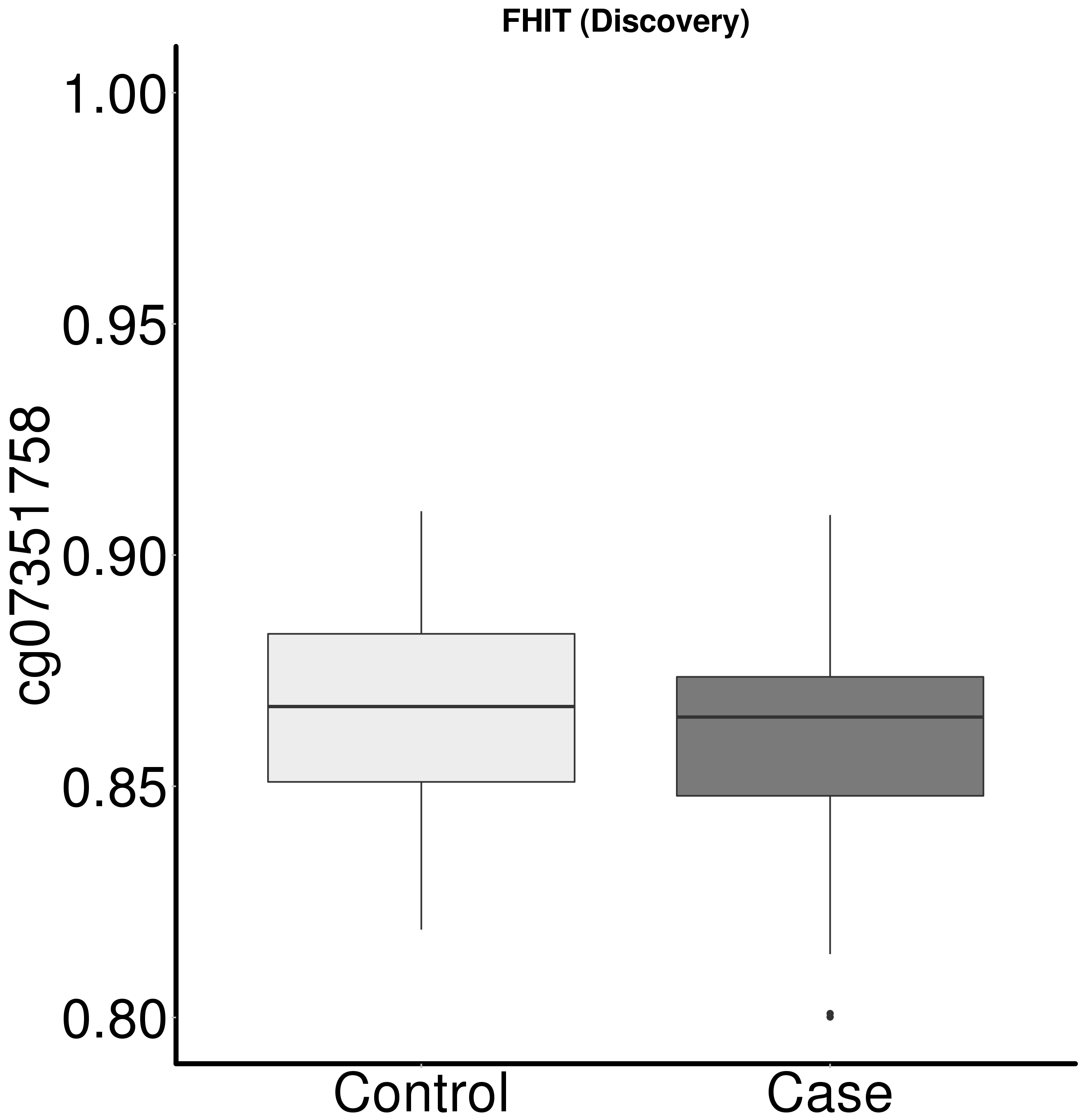
**

**
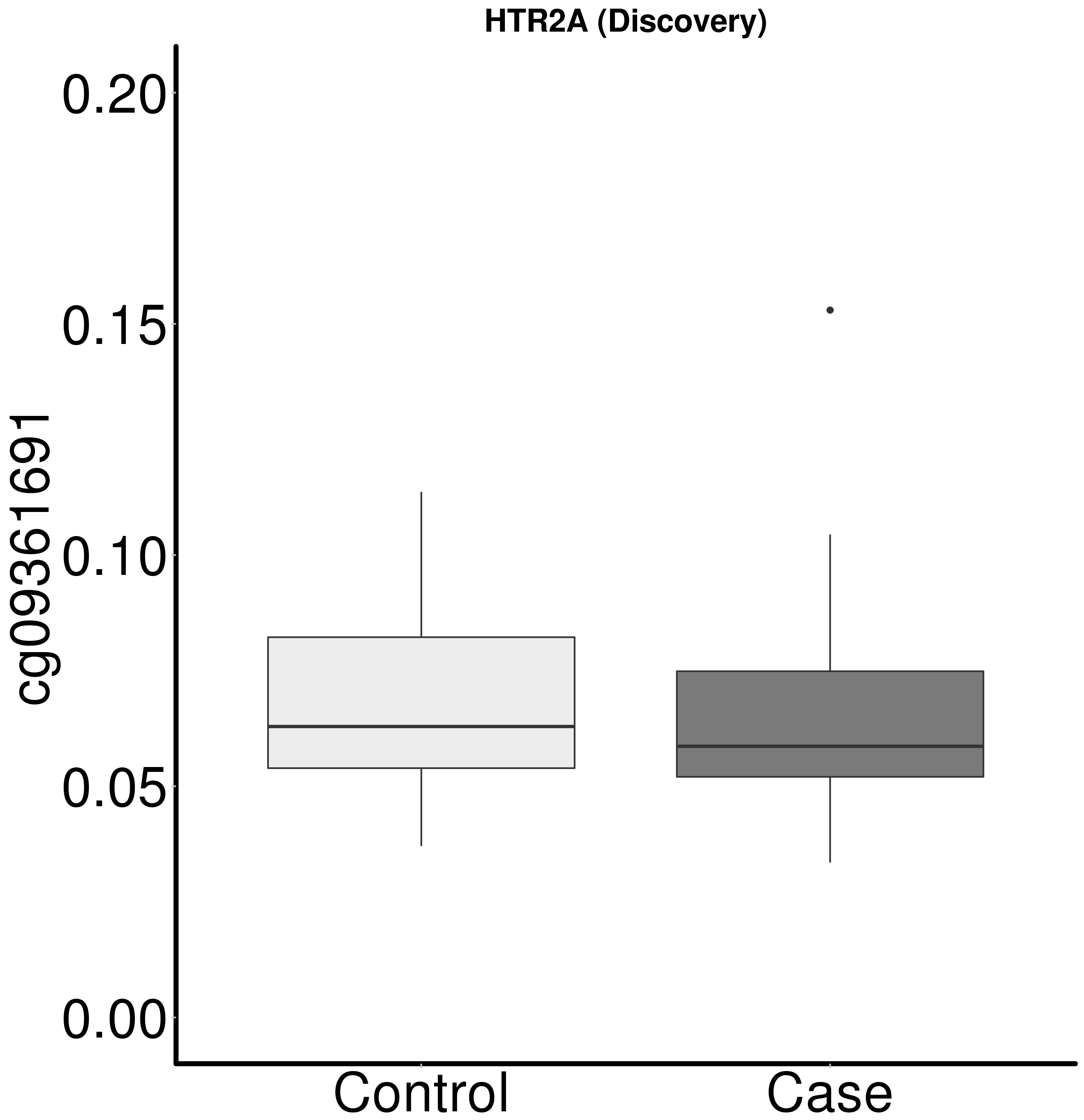

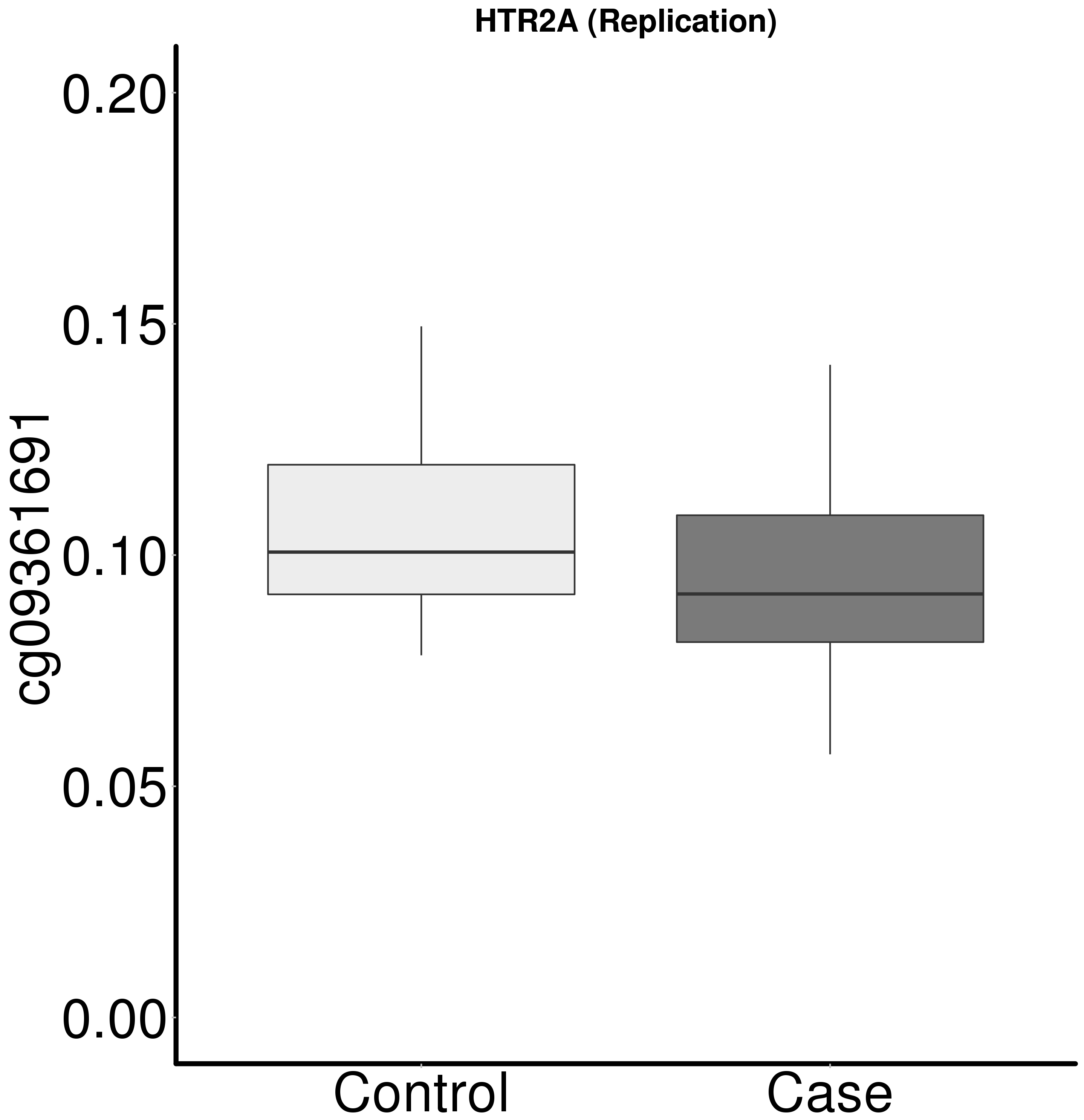
**

**Figure S7:**

Violin plots of ∆-age by case-control status in the replication sample. From the left: whole sample (P=0.282), males only (P=0.467), and females only (P=0.402).

**
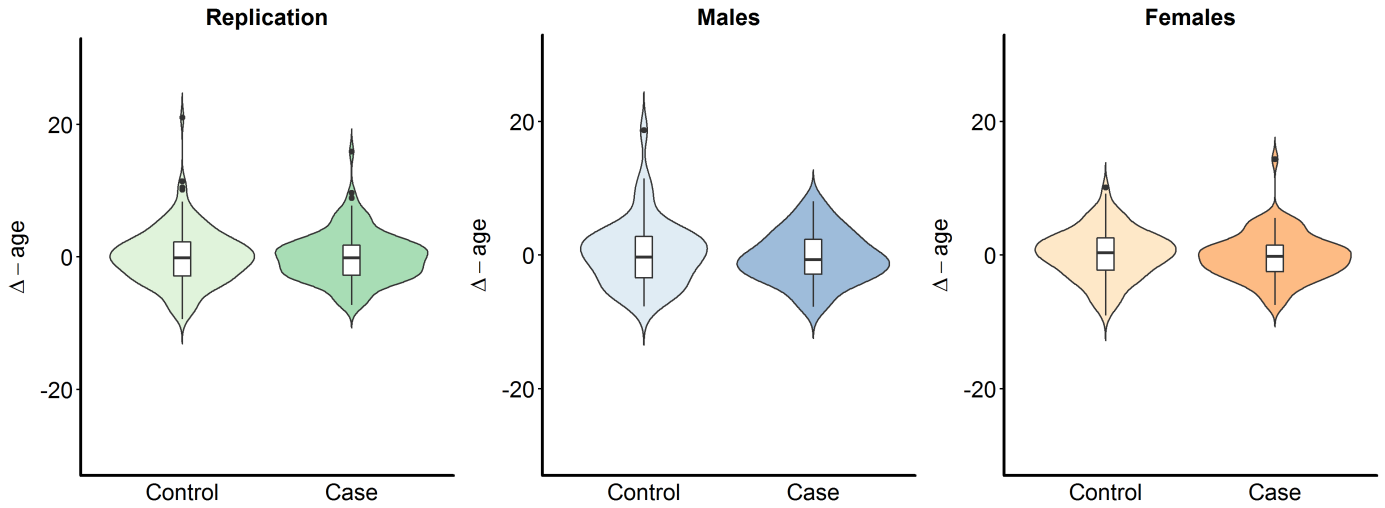
**

**Figure S8: HECA Gene Expression in Tissues**

Expression values are shown in RPKM (Reads Per Kilobase of transcript per Million mapped reads). Box plots are shown as median and 25th and 75th percentiles; points are displayed as outliers if they are above or below 1.5 times the interquartile range. Data Source: GTEx Analysis Release V6p (dbGaP Accession phs000424.v6.p1) (<http://www.gtexportal.org/home/>)

**

**

**
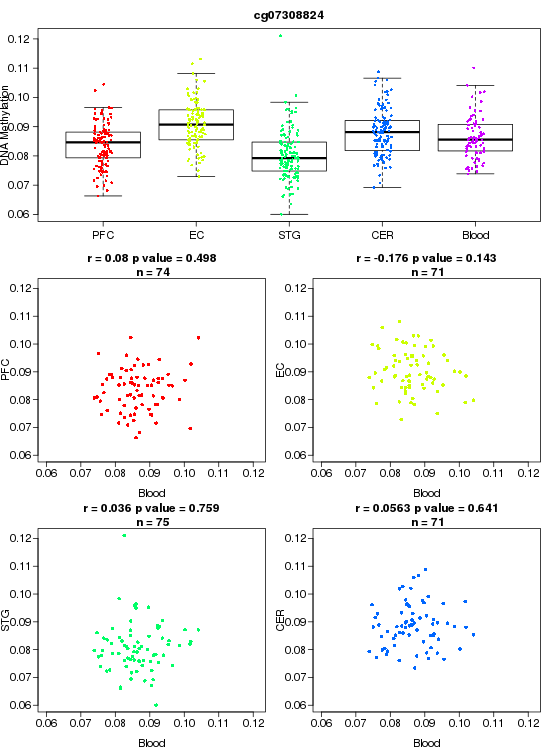
Figure S9:** Correlation of HECA methylation levels at the cg07308824 locus in whole blood and four brain regions in a linear regression model. The top panel is a boxplot with the DNA methylation levels per tissue type. Upper left panel: prefrontal cortex (PFC); lower left panel: superior temporal gyrus (STG); upper right panel: entorhinal cortex (EC); lower right panel: cerebellum (CER) (http://epigenetics.iop.kcl.ac.uk/bloodbrain/).
